# The addition of vorinostat to lenalidomide maintenance for patients with newly diagnosed multiple myeloma of all ages: results from ‘Myeloma XI’, a multicentre, open‐label, randomised, phase III trial

**DOI:** 10.1111/bjh.18600

**Published:** 2022-12-21

**Authors:** Matthew W. Jenner, Charlotte Pawlyn, Faith E. Davies, Tom Menzies, Anna Hockaday, Catherine Olivier, John R. Jones, Kamaraj Karunanithi, Jindriska Lindsay, Bhuvan Kishore, Gordon Cook, Mark T. Drayson, Martin F. Kaiser, Roger G. Owen, Walter Gregory, David A. Cairns, Gareth J. Morgan, Graham H. Jackson, for the UK National Cancer Research Institute (NCRI) Haemato‐oncology Clinical Studies Group

**Affiliations:** ^1^ University Hospital Southampton NHS Foundation Trust Southampton UK; ^2^ The Institute of Cancer Research London UK; ^3^ The Royal Marsden NHS Foundation Trust London UK; ^4^ Perlmutter Cancer Center, NYU Langone Health New York USA; ^5^ Cancer Research UK Clinical Trials Unit, Leeds Institute of Clinical Trials Research University of Leeds Leeds UK; ^6^ Eastbourne District General Hospital Eastbourne UK; ^7^ Brighton and Sussex Medical School University of Sussex Sussex UK; ^8^ Kings College Hospital NHS Foundation Trust London UK; ^9^ University Hospitals of North Midlands NHS Trust Stoke‐on‐Trent UK; ^10^ East Kent Hospitals University NHS Foundation Trust Canterbury UK; ^11^ University Hospitals Birmingham NHS Foundation Trust Birmingham UK; ^12^ Leeds Cancer Centre Leeds Teaching Hospitals Trust Leeds UK; ^13^ Institute of Immunology and Immunotherapy University of Birmingham Birmingham UK; ^14^ Haematological Malignancy Diagnostic Service (HMDS), Leeds Cancer Centre Leeds Teaching Hospitals Trust Leeds UK; ^15^ Department of Haematology Newcastle University Newcastle UK

**Keywords:** lenalidomide, maintenance combinations, myeloma, vorinostat

## Abstract

Lenalidomide is an effective maintenance agent for patients with myeloma, prolonging first remission and, in transplant eligible patients, improving overall survival (OS) compared to observation. The ‘Myeloma XI’ trial, for newly diagnosed patients, aimed to evaluate whether the addition of the histone deacetylase inhibitor vorinostat to the lenalidomide maintenance backbone could improve outcomes further. Patients included in this analysis were randomised to maintenance therapy with lenalidomide alone (10 mg/day on days 1–21 of each 28‐day cycle), or in combination with vorinostat (300 mg/day on day 1–7 and 15–21 of each 28‐day cycle) with treatment continuing until unacceptable toxicity or progressive disease. There was no significant difference in median progression‐free survival between those receiving lenalidomide‐vorinostat or lenalidomide alone, 34 and 40 months respectively (hazard ratio [HR] 1.18, 95% confidence interval [CI] 0.96–1.44, *p* = 0.109). There was also no significant difference in median OS, not estimable and 75 months respectively (HR 0.99, 95% CI 0.76–1.29, *p* = 0.929). Subgroup analysis demonstrated no statistically significant heterogeneity in outcomes. Combination lenalidomide‐vorinostat appeared to be poorly tolerated with more dose modifications, fewer cycles of maintenance therapy delivered and higher rates of discontinuation due to toxicity than lenalidomide alone. The trial did not meet its primary end‐point, there was no benefit from the addition of vorinostat to lenalidomide maintenance.

## INTRODUCTION

The clinical outcomes for patients with multiple myeloma have improved significantly in the last decade. However, nearly all patients will eventually relapse and although many can be retreated successfully at relapse, each remission is associated with diminishing depth and duration of response.^1^ The aim of a successful maintenance strategy is to extend of the first remission period with no adverse impact on subsequent treatment. It must also be a conveniently delivered therapy with manageable toxicity such that it does not impair quality of life or the ability to deliver concomitant medications.

Previous results of the UK National Cancer Research Institute (NCRI) Myeloma XI study have shown that the immunomodulatory agent lenalidomide can improve progression‐free survival (PFS) and overall survival (OS) in transplant‐eligible (TE) patients with newly diagnosed multiple myeloma, and PFS in transplant‐ineligible (TNE) patients, with manageable toxicities.^2^ These findings are supported by the results of other contemporaneous lenalidomide studies, including a meta‐analysis.^3^, ^4^ Going forward it is important to address whether these results can be improved further by the addition of a second synergistic agent given in combination, particularly for patients with high‐risk myeloma.

Vorinostat (suberanilohydroxamic acid [SAHA]) is a histone deacetylase (HDAC) inhibitor that inhibits the enzymatic activity of histone deacetylases HDAC1, HDAC2 and HDAC3 (Class 1) and HDAC6 (Class 2), leading to the removal of acetyl groups from the lysine residues of proteins including histones and transcription factors.^5^ Exposure of cell lines and primary human myeloma cells both in vitro and in vivo results in anti‐proliferative and pro‐apoptotic effects. Several trials of vorinostat in patients with myeloma have demonstrated clinical activity in combination with either lenalidomide^6^ or bortezomib^7^ but single‐agent activity was not established. In this study, using an adaptive design, we examined the use of maintenance lenalidomide‐vorinostat in comparison to lenalidomide alone.

## METHODS

The Myeloma XI trial is a phase III, multicentre, open‐label, parallel‐group, randomised controlled trial. Across two pathways, for TE and TNE patients, 4420 newly diagnosed, symptomatic, patients with myeloma were recruited. A number of primary outcomes have already been reported.^2^, ^8^, ^9^ Details of eligibility for the trial overall are available (Supplementary Methods); the trial was designed to be an all‐comers study with few exclusion criteria. The trial was performed in accordance with the Declaration of Helsinki 1996, and the study was approved by the national ethics review board (National Research Ethics Service, London, UK), institutional review boards of the participating centres, and the competent regulatory authority (Medicines and Healthcare Products Regulatory Agency, London, UK). All patients provided written informed consent. The trial was registered with the European Union Drug Regulating Authorities Clinical Trials Database (EudraCT number, 2009‐010956‐93) and the International Standard Randomised Controlled Trial Number registry (ISRCTN49407852).

### Study design and treatment

The trial design included an intensive treatment pathway for TE patients and a non‐intensive treatment pathway for TNE patients. Patients received a minimum of four cycles (TE) or six cycles (TNE) of immunomodulatory agent‐based induction therapy in the absence of progressive disease (PD), and treatment continued until maximum response was achieved.

Additional induction intensification therapy was administered to patients with a suboptimal response using a response‐adapted approach: patients with stable disease (SD) after induction therapy or those with PD at any time during induction therapy received a maximum of eight cycles of cyclophosphamide, bortezomib, and dexamethasone (CVD); patients with a minimal response (MR) or partial response (PR) were randomised (1:1) to CVD or no CVD. Patients with very good PR (VGPR) or complete response (CR) received no additional therapy.

At maximum response following induction, or induction intensification if given, eligible patients were randomised to maintenance therapy with lenalidomide alone (10 mg/day on days 1–21 of each 28‐day cycle), or in combination with vorinostat (300 mg/day on day 1–7 and 15–21 of each 28‐day cycle) until unacceptable toxicity or PD, or to observation without maintenance therapy. Patients were excluded from maintenance randomisation if they did not respond to lenalidomide‐based induction, had no response to any prior study treatment, had PD or relapsed after achieving CR.

The analyses presented here focus on outcomes according to lenalidomide and combination lenalidomide‐vorinostat (R vs. RZ) among all patients. Further details on the dose and schedule of all study treatments are provided in Table S2.

### Study end‐points

The co‐primary end‐points were PFS and OS. Secondary end‐points included PFS Two (PFS2), response and safety. For time‐to‐event end‐points, the relative difference in hazard was quantified with a hazard ratio (HR), where a HR <1 indicates a benefit for combination lenalidomide‐vorinostat over lenalidomide. End‐point definitions and further details of the statistical analysis are included in the Supplementary Methods.

### Statistical analysis

The hypothesis of the maintenance randomisation presented here was that combination lenalidomide‐vorinostat treatment could improve PFS and OS compared with lenalidomide in adult patients with multiple myeloma. For PFS, the trial was designed to demonstrate a 7.3‐month increase in median PFS in the combination lenalidomide‐vorinostat group (median 34 months) compared with the lenalidomide group (median 26.7 months, HR 0.79) when 539 PFS events had been observed. For OS, it was designed to demonstrate a 10% increase in 5‐year OS in the combination lenalidomide‐vorinostat group (65% at 5 years) compared with the lenalidomide group (55% at 5 years, HR 0.72) when 285 OS events had been observed. Each of these calculations assumed the time to event was exponentially distributed and that recruitment would last 3.25 years with 4 years of further follow‐up, a two‐sided 5% significance level, and 80% or 78% power respectively. A minimum recruitment target of 707 patients randomly assigned to (1:1) combination lenalidomide‐vorinostat and lenalidomide was specified, allowing for a 2% dropout.

Formal interim analyses were prespecified in the study protocol for harm considering PFS when ≥25% of required events had been observed (≥130 progressions or deaths) following the method suggested by Freidlin et al.^10^ where if the lower confidence interval (CI) bound of the HR is >1 (vorinostat is harmful) then the vorinostat‐containing treatment arm should be stopped; and for efficacy considering PFS and OS when ≥50% of required events had been observed (≥269 progressions or deaths and ≥143 deaths). To ensure an overall significance level of 5% would be maintained in the efficacy interim analysis, the O'Brien and Fleming alpha‐spending function (interim analysis bound 0.5%, final analysis bound 4.7%) was used.^11^ The bound for the efficacy interim analysis was advisory, with the decision to release results at the recommendation of the Independent Myeloma XI Data Monitoring and Ethics Committee (DMEC) and the Independent Myeloma XI Trial Steering Committee (TSC). The interim analysis for harm was done and presented to the DMEC on 1 October 2015 and their recommendations passed to the TSC on 4 October 2015, and the study continued without reporting the interim analysis. The interim analysis for efficacy was done and presented to the DMEC on 30 October 2017, where it was decided that, in the opinion of the committee, combination lenalidomide‐vorinostat treatment had negligible chance of showing superiority as compared to lenalidomide but may be contributing to lower delivery of lenalidomide and thus affecting the benefit seen with lenalidomide on PFS and OS for these patients. Their recommendations were passed to the TSC on 1 November 2017, and a change to the protocol was implemented through an Urgent Safety Measure with submission to the Medicines and Healthcare Products Regulatory Agency (MHRA) and National Research Ethics Service on 2 November 2017. Participating centres were notified on 3 November 2017 to stop treatment with vorinostat in the combination lenalidomide‐vorinostat group. The key analysis of primary end‐points that oversight committees used in making this decision is shown in Figure S1.

The data cut‐off date for this analysis was 31 May 2019. Presented here are the results of the co‐primary and secondary end‐points, and pre‐specified subgroup analysis for the maintenance randomisation comparing combination lenalidomide‐vorinostat and lenalidomide. Other exploratory end‐points will be reported elsewhere. Efficacy analyses were done by intention to treat, including all patients randomly assigned to combination lenalidomide‐vorinostat or lenalidomide. The safety population included all patients who received at least one dose of maintenance therapy. All reported *p* values are two‐sided and considered significant at an overall significance level of 5%. Further trial information is detailed in the Supplementary Methods.

For the co‐primary end‐points, summaries of time‐to‐event per treatment group were estimated using the Kaplan–Meier method. Comparisons between the allocated groups were made using the Cox proportional hazards model stratified by the minimisation stratification factors, excluding centre, and to estimate HRs and 95% CIs. Similar methods were used to assess the secondary outcome of PFS2. Subgroup analysis was prespecified for the presence or absence of individual adverse cytogenetic abnormalities, cytogenetic risk status, and induction and consolidation treatment (CVD intensification and transplantation eligibility). A likelihood ratio test (LRT) for heterogeneity of treatment effect using Cox models identical to those used for the main analysis was performed, with the inclusion of terms for the subgroup in question and the appropriate interaction term. The reported test for heterogeneity for subgroup analysis corresponds to a one degree of freedom test for two category subgroups and a two degrees of freedom test for three category subgroups. An exploratory analysis with a saturated three‐way interaction model for maintenance allocation, transplant eligibility and induction allocation was undertaken, which was assessed using similar LRT.

Toxicity is summarised in terms of adverse events, descriptively. Cumulative incidence function curves for time to second primary malignancies (SPM) was calculated by non‐parametric maximum likelihood estimation. Fine and Gray competing risks regression was used to compare the hazard of SPM by allocated treatment, adjusting for the minimisation stratification factors, with unrelated deaths specified as a competing risk.

Post hoc exploratory analyses undertaken were the effect on PFS and OS, and PFS2 of the subgroups sex, age, disease stage according to the International Staging System (ISS), and response at start of maintenance; analysis of the effect of induction or intensification treatment.

The Statistical Analysis System (SAS; version 9.4), Stata/IC (version 14.2), and R (version 3.2.3) were used for statistical analyses. This report presents final exploratory results with long‐term follow‐up.

## RESULTS

### Patients

**FIGURE 1:**
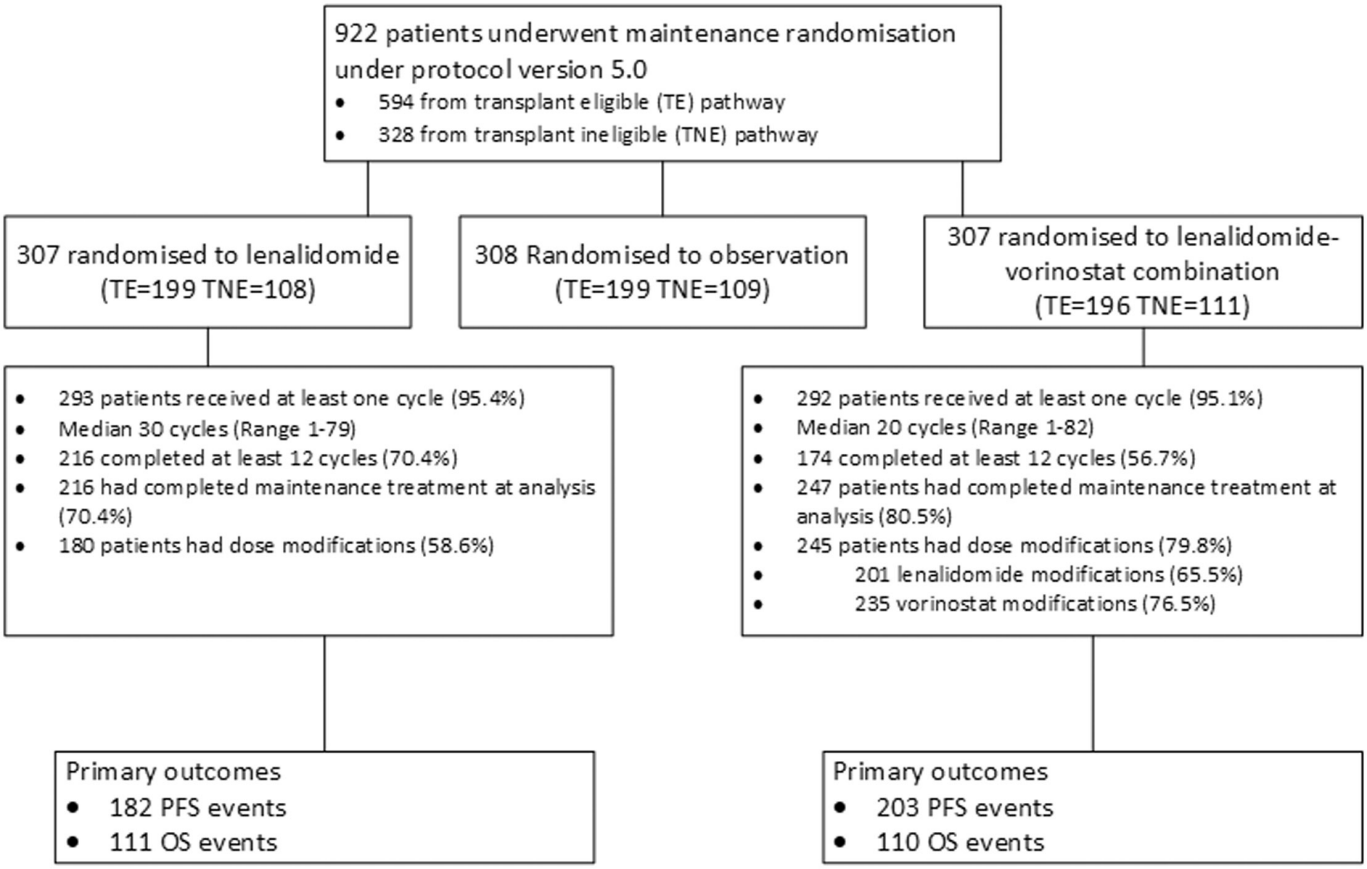
Consolidated Standards of Reporting Trials (CONSORT) diagram for the Myeloma XI trial maintenance randomisation (protocol version 5.0). PFS, progression‐free survival; OS, overall survival

Between June 2012 and June 2015, 614 patients underwent the maintenance randomisation under protocol version 5.0 (Figure 1), 307 to lenalidomide and 307 to combination lenalidomide‐vorinostat. Baseline characteristics were balanced between the two treatment groups (Table 1). Overall, the median (range) patient age was 66 (29–86) years, 286 (42.7%) of the patients were aged >65 years, 457 (74.4%) of the patients had a World Health Organization (WHO) performance status of ≤1 and 152 (24.8%) had ISS Stage III disease. In all, 395 patients were randomised following autologous stem cell transplantation in the TE pathway and 219 within the TNE pathway.

**TABLE 1:** Patient characteristics according to maintenance regimen in protocol version 5 (intention‐to‐treat population)

Characteristic	Lenalidomide maintenance (*n* = 307)	Lenalidomide and vorinostat maintenance (*n* = 307)
Age, years, median (range)	66.0 (29.0–85.0)	66.0 (35.0–86.0)
Age, years, *n* (%)		
≤65	168 (54.7)	160 (52.1)
>65	139 (45.3)	147 (47.9)
Sex, *n* (%)		
Male	191 (62.2)	171 (55.7)
Female	116 (37.8)	136 (44.3)
Ethnicity, *n* (%)		
White	288 (93.8)	285 (92.8)
Black (e.g., Black Caribbean, Black African)	7 (2.3)	6 (2.0)
Asian (e.g., Indian, Pakistani, Bangladeshi)	4 (1.3)	6 (2.0)
Other	5 (1.6)	6 (2.0)
Unknown	3 (1.0)	4 (1.3)
WHO performance status, *n* (%)		
0	117 (38.1)	105 (34.2)
1	114 (37.1)	121 (39.4)
2	44 (14.3)	55 (17.9)
≥3	17 (5.5)	12 (3.9)
Not available	15 (4.9)	14 (4.6)
Ig subtype, *n* (%)		
IgG	187 (60.9)	184 (59.9)
IgA	73 (23.8)	79 (25.7)
IgM	2 (0.7)	1 (0.3)
IgD	2 (0.7)	3 (1.0)
Light chain only	39 (12.7)	38 (12.4)
Non‐secretor	4 (1.3)	1 (0.3)
Not available	0	1 (0.3)
ISS Stage, *n* (%)		
I	82 (26.7)	82 (26.7)
II	125 (40.7)	125 (40.7)
III	76 (24.8)	76 (24.8)
Not available	24 (7.8)	24 (7.8)
Creatinine, μmol/L, median (range)	85.0 (36.0–494.0)	83.0 (34.0–444.0)
Unknown, *n*	2	0
Lactate dehydrogenase, iu/L, median (range)	253.0 (3.0–3205.0)	262.0 (57.0–1884.0)
Unknown, *n*	72	62
Transplantation eligibility and induction regimen		
Transplantation eligible	199 (64.8)	196 (63.9)
CTD	94 (30.6)	93 (30.3)
CRD	105 (34.2)	103 (33.6)
Transplantation ineligible	108 (35.2)	111 (36.2)
Attenuated CTD	49 (16.0)	50 (16.3)
Attenuated CRD	59 (19.2)	61 (19.9)
CVD intensification, *n* (%)		
Randomised to no CVD after PR/MR	33 (10.7)	32 (10.4)
Randomised to CVD after PR/MR	30 (9.8)	29 (9.4)
Received CVD after SD/PD	3 (1.0)	2 (0.7)
Cytogenetic data available, *n* (%)	140 (45.6)	139 (45.3)
gain(1q) detected^c^	48 (34.3)	36 (25.9)
t(4, 14) detected^c^	15 (10.7)	14 (10.1)
t(14, 16) detected^c^	8 (5.7)	2 (1.4)
t(14,20) detected^c^	3 (2.1)	1 (0.7)
del(17p) detected^c^	13 (9.3)	14 (10.1)
Cytogenetic risk category, *n* (%)		
Standard^c^	73 (52.1)	87 (62.6)
High^a^ ^,^ ^c^	50 (35.7)	38 (27.3)
Ultra‐high^b^ ^,^ ^c^	17 (12.1)	14 (10.1)
Response at maintenance randomisation, *n* (%)		
CR or VGPR	247 (80.5)	241 (78.5)
CR	64 (20.8)	53 (17.3)
CR (w/o BM)	97 (31.6)	113 (36.8)
VGPR	86 (28.0)	75 (24.4)
PR or MR	53 (17.3)	60 (19.5)
PR	50 (16.3)	55 (17.9)
MR	3 (1.0)	5 (1.6)
SD or PD	3 (1.0)	3 (1.0)
SD	0	1 (0.3)
PD	3 (1.0)	2 (0.7)
Not available	4 (1.3)	3 (1.0)

Abbreviations: C, cyclophosphamide; D, dexamethasone; Ig, immunoglobulin; ISS, International Staging System; R, lenalidomide; T, thalidomide; WHO, World Health Organization; CR, complete response; VGPR, very good partial response; PR, partial response; MR, minimal response; SD, stable disease; PD, progressive disease.

^a^ High risk defined as the presence of any one of t(4;14), t(14;16), t(14;20), del(17p) or gain(1q).

^b^ Ultra‐high risk defined as the presence of more than one high risk lesion.

^c^ Percentage of those with cytogenetic data available.

### Impact of maintenance treatment

**FIGURE 2:**
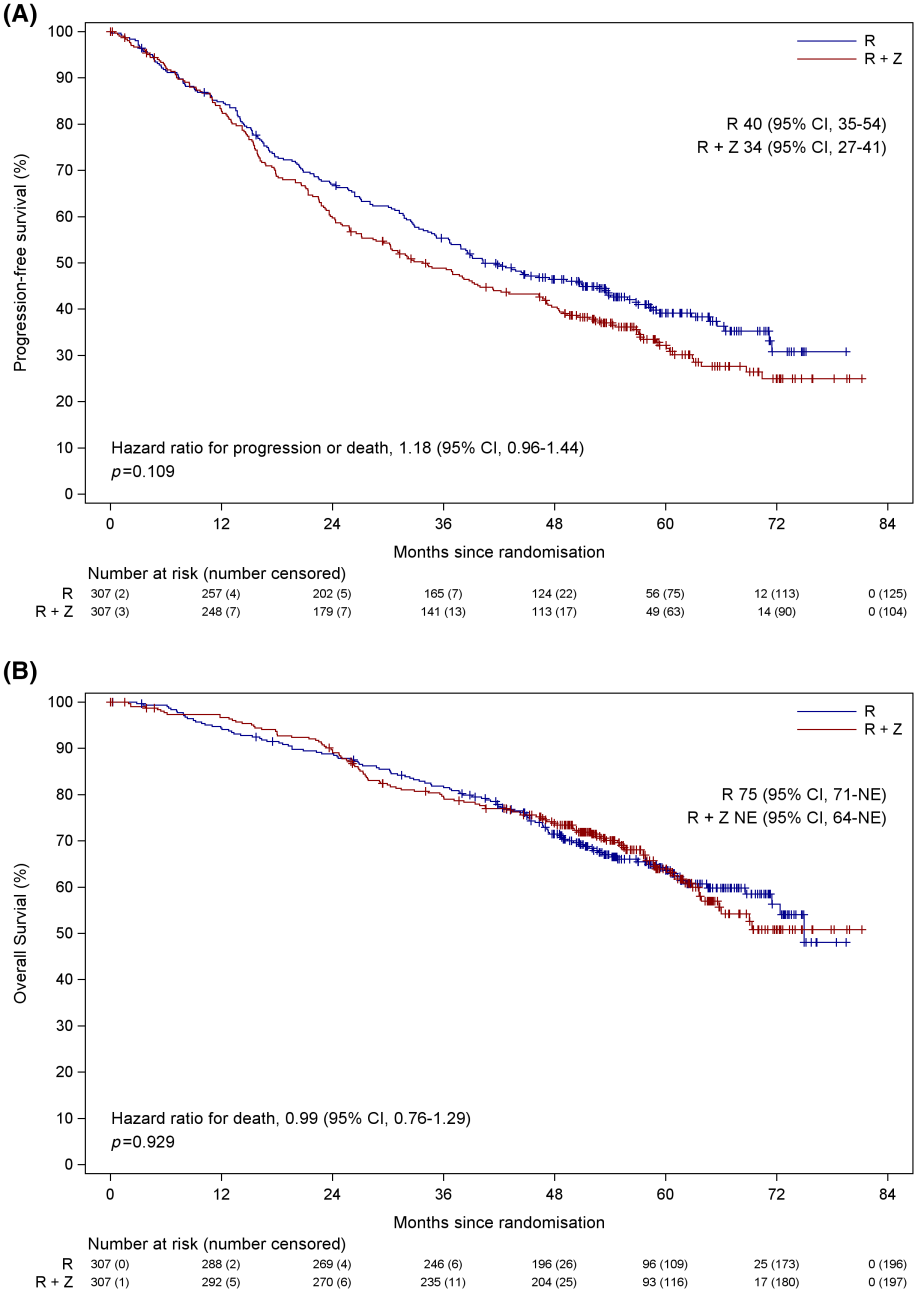
PFS and OS primary end‐point analysis. (**A**) PFS by randomised treatment. (**B**) OS by randomised treatment. CI, confidence interval; NE, not estimable; OS, overall survival; PFS, progression‐free survival; R, lenalidomide; R + Z, combination lenalidomide‐vorinostat

At the time of this analysis the median (interquartile range [IQR]) follow‐up from randomisation was 60 (53–68) months, 203 instances of progression or death had occurred in the combination lenalidomide‐vorinostat group and 182 in the lenalidomide group. A significant difference in the median (95% CI) PFS was not identified, 34 (27–41) months and 40 (35–54) months respectively (HR 1.18, 95% CI 0.96–1.44, *p* = 0.109) (Figure 2A). The difference in median PFS in favour of lenalidomide of 6 months was contrary to the expected minimum clinically relevant difference of 7.3 months in favour of combination lenalidomide‐vorinostat. In the combination lenalidomide‐vorinostat group 110 deaths were seen compared to 111 in the lenalidomide group with no significant difference in median (95% CI) OS, not estimable (NE) (64–NE) months and 75 (71–NE) months respectively (HR 0.99, 95% CI 0.76–1.29, *p* = 0.929) (Figure 2B). The median (95% CI) percentage of patients alive at 5 years after maintenance randomisation was 63.9% (57.9%–69.9%) in the combination lenalidomide‐vorinostat group and 63.6% (57.8%–69.5%) in the lenalidomide group. The difference in 5‐year OS in favour of combination lenalidomide‐vorinostat of 0.3% points was not as large as the expected minimum clinically relevant difference of 5% points. These results are similar to those seen at the time of the interim analysis (Figure S1).

**FIGURE 3:**
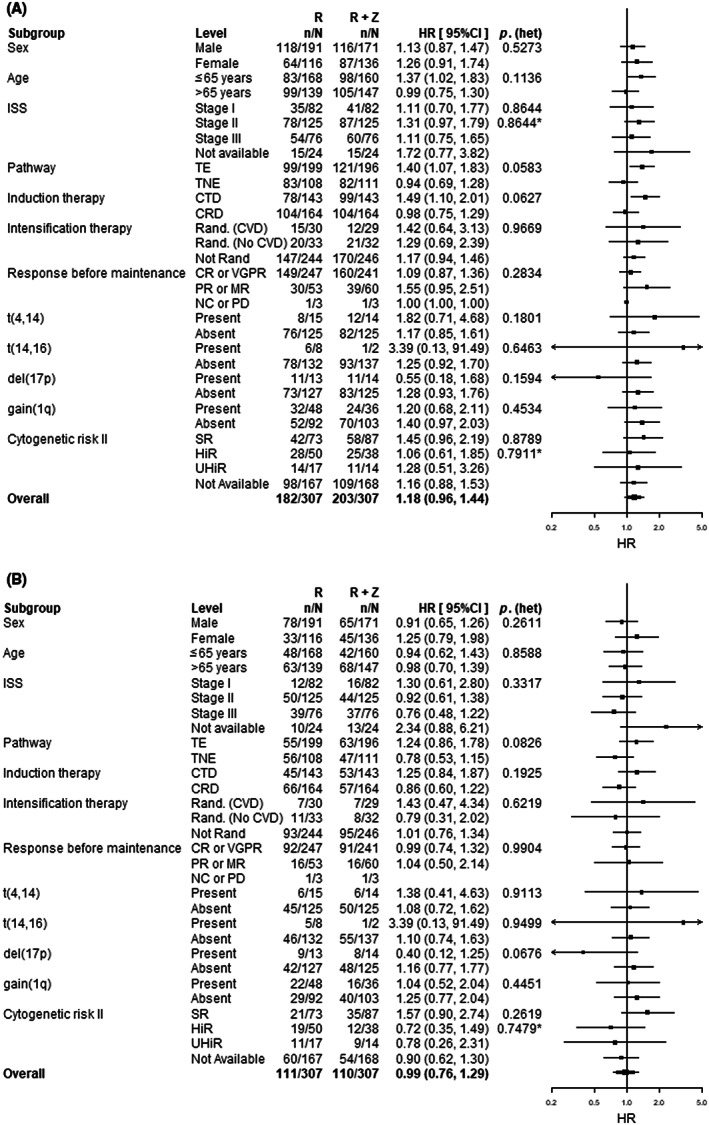
Subgroup analysis for primary end‐points. (**A**) Forest plot of the subgroup analysis for PFS. (**B**) Forest plot of the subgroup analysis for OS. The black squares and horizontal lines represent the hazard ratio (HR) and the associated 95% confidence interval (95% CI) of the hazard of progression or death (PFS) and hazard of death (OS) in the combination lenalidomide‐vorinostat (R + Z) group compared to the lenalidomide (R) group, *p*(het) represents the *p* value from the likelihood ratio test assessing heterogeneity of treatment effect between subgroups. CRD, cyclophosphamide, lenalidomide and dexamethasone; CTD, cyclophosphamide, thalidomide and dexamethasone; CVD, cyclophosphamide, bortezomib, and dexamethasone; HiR, high‐risk; ISS, International Staging System; NE, not estimable; SR, standard risk; TE, transplant eligible; TNE, transplant ineligible; UHiR; ultra‐high‐risk. *Likelihood ratio test for heterogeneity of effect among patients with subgroup data available

Subgroup analysis identified no statistically significant heterogeneity in primary outcomes (Figure 3A,B). There was weak evidence of heterogeneity of PFS in relation to age (*p* = 0.1136), potentially representing an association with transplant eligibility (*p* = 0.0583) and the induction treatment used (*p* = 0.0627). A similar association was seen for OS, for transplant eligibility (*p* = 0.0826) and allocated induction treatment (*p* = 0.1925). For the TE patients, the use of lenalidomide‐vorinostat was associated with a significantly impaired median PFS (lenalidomide 58 months vs. combination lenalidomide‐vorinostat 39 months; HR 1.40, 95% CI 1.07–1.83, *p* = 0.013; Figure S2A), but this difference was not seen for OS (lenalidomide NE months vs. combination lenalidomide‐vorinostat NE months; HR 1.24, 95% CI 0.86–1.78, *p* = 0.245; Figure S2B). For the TNE patients there was no difference in median PFS (lenalidomide 26 months vs. combination lenalidomide‐vorinostat 26 months; HR 0.94, 95% CI 0.69–1.28, *p* = 0.687; Figure S2C), or OS (lenalidomide 61 months vs. combination lenalidomide‐vorinostat 64 months; HR 0.78, 95% CI 0.53–1.15, *p* = 0.202; Figure S2D). For patients allocated to induction with cyclophosphamide, thalidomide and dexamethasone (CTD[a]) combination lenalidomide‐vorinostat was associated with a significantly inferior median PFS (lenalidomide 45 months vs. combination lenalidomide‐vorinostat 30 months; HR 1.50, 95% CI 1.11–2.02, *p* = 0.008; Figure S3A), but not OS (lenalidomide NE months vs. combination lenalidomide‐vorinostat 69 months; HR 1.25, 95% CI 0.84–1.87, *p* = 0.267; Figure S3B). This may have been related to the lower response rate in the CTD(a) arms as patients having a PR or an MR prior to maintenance had a trend towards inferior outcomes with lenalidomide‐vorinostat (Figure S4A,B). For patients allocated to cyclophosphamide, lenalidomide and dexamethasone (CRD[a]) induction lenalidomide‐vorinostat was not associated with any difference in median PFS (lenalidomide 38 months vs. combination lenalidomide‐vorinostat 37 months; HR 1.01, 95% CI 0.77, 1.33, *p* = 0.920; Figure S3C), or OS (lenalidomide 71 months vs. combination lenalidomide‐vorinostat NE months; HR 0.87, 95% CI 0.61–1.24, *p* = 0.435; Figure S3D). An exploratory analysis testing all two‐ and three‐way interactions between maintenance allocation, transplant‐eligibility and allocated induction treatment did not identify significant statistical interactions (LRT *p* = 0.146 on four degrees of freedom). There was no evidence of significant heterogeneity of treatment effect with respect to cytogenetic risk status groups, although there was a trend towards a benefit for the use of lenalidomide‐vorinostat for those patients with del(17p); however, there were only a small number of patients with this feature (Figure 3A,B, Figure S5).

**FIGURE 4:**
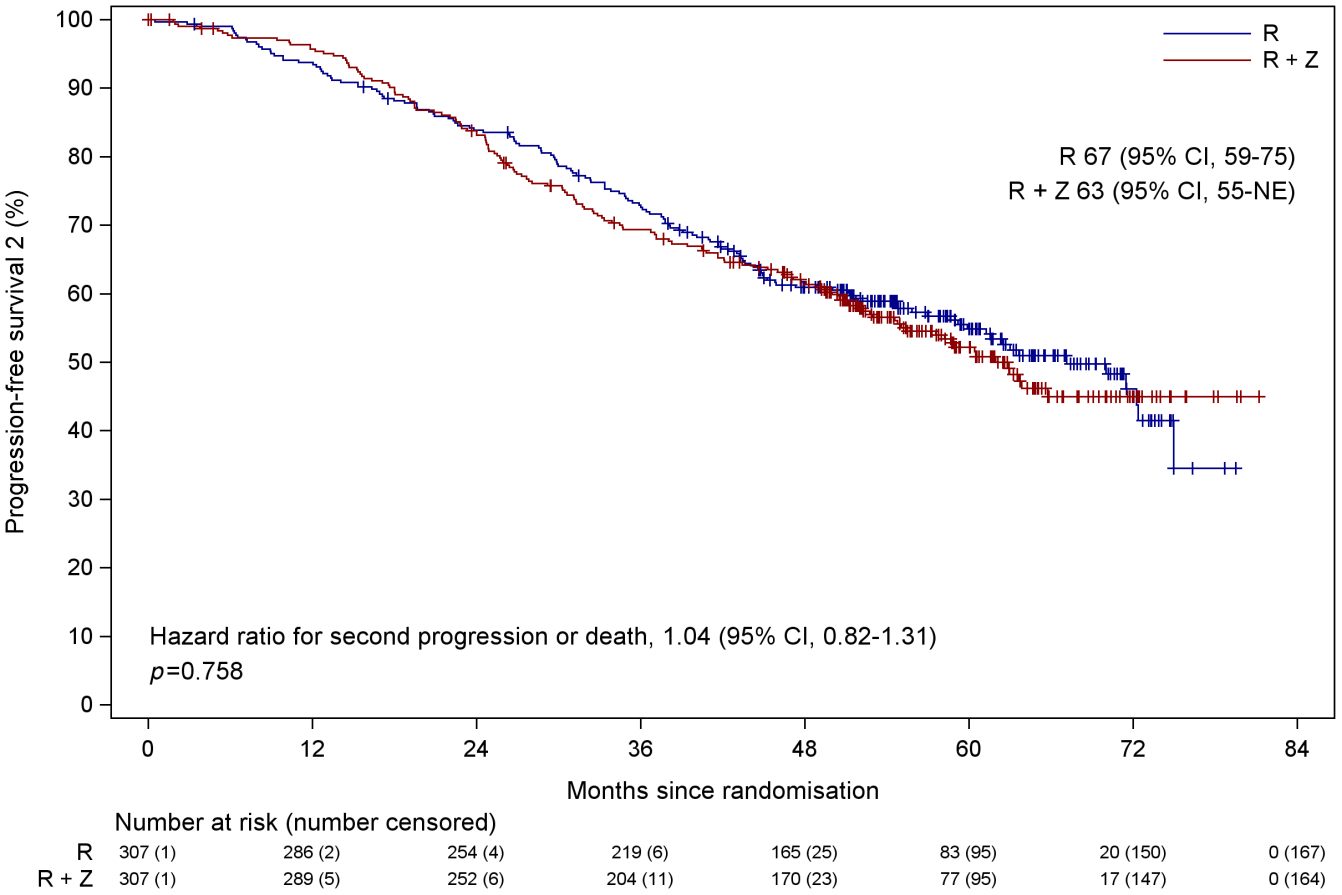
Progression‐free survival two (PFS2) secondary end‐point analysis by randomised treatment. CI, confidence interval; NE, not estimable; R, lenalidomide; R + Z, combination lenalidomide‐vorinostat

Second progression or deaths occurred in 143 patients in the combination lenalidomide‐vorinostat group compared to 140 in the lenalidomide group with no significant difference in median PFS2, 63 and 67 months respectively (HR 1.04, 95% CI 0.82–1.31, *p* = 0.758) (Figure 4). For TE patients the combination lenalidomide‐vorinostat did not show a significant difference in median PFS2 (lenalidomide 77 months vs. combination lenalidomide‐vorinostat 62 months; HR 1.25, 95% CI 0.92–1.71, *p* = 0.158; Figure S6A). This was also the case for TNE patients although with a reversed trend (lenalidomide 46 months vs. combination lenalidomide‐vorinostat 55 months; HR 0.82, 95% CI 0.57–1.17, *p* = 0.265; Figure S6B).

### Treatment delivered and side‐effects

The median (range) number of cycles of maintenance therapy delivered was 20 (one–82) for the combination lenalidomide‐vorinostat and 30 (one–79) for lenalidomide with 174 (56.7%) and 216 (70.4%) completing at least 12 cycles respectively. Combination lenalidomide‐vorinostat was less well tolerated and was associated with more dose modifications. Dose modifications occurred in 180 (58.6%) patients in the lenalidomide group and 245 patients (79.8%) in the combination lenalidomide‐vorinostat group. In the combination lenalidomide‐vorinostat group 201 patients (65.5%) had a modification to lenalidomide and 235 patients (76.5%) had a modification to vorinostat.

The median (IQR) percentage of minimum protocol lenalidomide dose delivered in the lenalidomide group was 78.2% (43.1%–100.0%) and in the lenalidomide‐vorinostat combination group was 54.6% (18.9%–93.8%). The lowest percentage of minimum protocol dose delivered was in the patients receiving CTD(a) induction in both groups (Table S3). The median (IQR) percentage of the minimum protocol dose delivered for vorinostat was 30.2% (6.1%–67.7%). The rate of discontinuation of maintenance therapy due to toxicity was higher in the combination lenalidomide‐vorinostat group compared to the lenalidomide group (25.4% and 12.4% respectively, Table S4). The rate of discontinuation due to unacceptable toxicity in the combination group following induction treatment with CTD(a) was higher than with CRD(a), at 26.9% and 22.3% respectively for TE, and 34.0% and 21.3% respectively for TNE (Table S4).

Adverse events were assessed in the 585 patients who completed at least one dose of study drug (Table 2). Grade 3 and 4 haematological adverse reactions were more frequent in the lenalidomide‐vorinostat group than in the lenalidomide group: neutropenia in 123 (42.1%) patients compared to 102 patients (34.9%); thrombocytopenia in 39 (13.4%) patients compared to 18 patients (6.2%); and anaemia in 17 (5.8%) patients compared to nine patients (3.1%). More patients in the combination lenalidomide‐vorinostat group had Grade 1–2 anorexia (13.7% vs. 6.8%) and nausea (22.9% vs. 16.0%), although there was no difference in vomiting. There was no difference in other commonly experienced toxicities including fatigue, infections, or rashes. Lower rates of arthralgia and back pain were noted with lenalidomide‐vorinostat (Grade 1–2 arthralgia 9.2% vs. 18.1%, Grade 1–2 back pain 14.4% vs. 22.9%). A Grade 3 and 4 pulmonary embolism occurred in five (1.7%) patients in the combination lenalidomide‐vorinostat group and two (0.6%) in the lenalidomide group. Fatal adverse events during maintenance were most commonly due to respiratory infection (0.3%) and sepsis (0.7%) in both groups. There was no difference in the 3‐year cumulative incidence of invasive SPM between combination lenalidomide‐vorinostat and lenalidomide maintenance treatment (3.6% vs. 2.5%; HR 0.82, 95% CI 0.45–1.48, *p* = 0.5099).

**TABLE 2:** Adverse events (safety population)

	Combination lenalidomide‐vorinostat, *n* (%)				Lenalidomide, *n* (%)			
	Grade 1–2	Grade 3	Grade 4	Grade 5	Grade 1–2	Grade 3	Grade 4	Grade 5
Neutrophil count decrease	106 (36.3)	109 (37.3)	14 (4.8)	0	130 (44.4)	91 (31.1)	11 (3.8)	0
Platelet count decrease	164 (56.2)	33 (11.3)	6 (2.1)	0	153 (52.2)	11 (3.8)	7 (2.4)	0
Anaemia	195 (66.8)	14 (4.8)	3 (1.0)	0	206 (70.3)	9 (3.1)	0	0
Peripheral motor neuropathy	40 (13.7)	1 (0.3)	0	0	41 (14.0)	1 (0.3)	0	0
Peripheral sensory neuropathy	86 (29.5)	2 (0.7)	0	0	109 (37.2)	2 (0.7)	0	0
Constipation	102 (34.9)	2 (0.7)	0	0	96 (32.8)	1 (0.3)	0	0
Pulmonary embolism	0	5 (1.7)	0	0	0	1 (0.3)	1 (0.3)	0
Other thrombosis/ embolism	0	0	1 (0.3)	0	3 (1.0)	1 (0.3)	0	0
Abdominal pain	15 (5.1)	3 (1.0)	0	0	16 (5.5)	0	0	0
Abnormal LFTs	1 (0.3)	1 (0.3)	0	0	0	0	0	0
Abscess	5 (1.7)	0	0	0	3 (1.0)	1 (0.3)	0	0
Acute kidney injury	1 (0.3)	2 (0.7)	1 (0.3)	1 (0.3)	4 (1.4)	0	0	0
ALT increased	14 (4.8)	8 (2.7)	0	0	9 (3.1)	2 (0.7)	1 (0.3)	0
ALP increased	7 (2.4)	1 (0.3)	0	0	5 (1.7)	2 (0.7)	0	0
Anorexia	40 (13.7)	1 (0.3)	0	0	20 (6.8)	0	0	0
Arthralgia	27 (9.2)	1 (0.3)	0	0	53 (18.1)	1 (0.3)	0	0
AST increased	0	1 (0.3)	0	0	0	1 (0.3)	0	0
Atrial fibrillation	1 (0.3)	1 (0.3)	0	0	1 (0.3)	0	0	0
Atrioventricular block complete	0	1 (0.3)	0	0	0	0	0	0
Back pain	42 (14.4)	1 (0.3)	0	0	67 (22.9)	1 (0.3)	0	0
Blood bilirubin increased	1 (0.3)	0	1 (0.3)	0	4 (1.4)	0	0	0
Bone pain	12 (4.1)	1 (0.3)	0	0	16 (5.5)	0	0	0
Cardiac disorders – other	0	1 (0.3)	0	0	3 (1.0)	0	0	0
Cataract	0	1 (0.3)	0	0	0	0	0	0
Cellulitis	2 (0.7)	1 (0.3)	0	0	5 (1.7)	1 (0.3)	0	0
Cholecystitis	0	0	0	0	1 (0.3)	1 (0.3)	0	0
Chronic kidney disease	1 (0.3)	3 (1.0)	0	0	2 (0.7)	2 (0.7)	0	0
Confusion	2 (0.7)	0	0	0	0	1 (0.3)	0	0
Cough	44 (15.1)	2 (0.7)	0	0	50 (17.1)	4 (1.4)	0	0
Creatinine increased	13 (4.5)	0	1 (0.3)	0	9 (3.1)	0	0	0
Dehydration	0	0	0	0	1 (0.3)	2 (0.7)	0	0
Dental abscess	1 (0.3)	1 (0.3)	0	0	2 (0.7)	0	0	0
Depression	5 (1.7)	1 (0.3)	0	0	15 (5.1)	1 (0.3)	0	0
Dizziness	12 (4.1)	0	0	0	20 (6.8)	1 (0.3)	0	0
Dysarthria	0	1 (0.3)	0	0	0	0	0	0
Dyspnoea (shortness of breath)	19 (6.5)	1 (0.3)	0	0	26 (8.9)	3 (1.0)	0	0
Ear and labyrinth disorders – other	0	0	0	0	2 (0.7)	1 (0.3)	0	0
Elevated CRP	4 (1.4)	1 (0.3)	0	0	6 (2.0)	1 (0.3)	0	0
Epistaxis	5 (1.7)	1 (0.3)	0	0	2 (0.7)	0	0	0
Fatigue/lethargy	114 (39.0)	5 (1.7)	0	0	115 (39.2)	2 (0.7)	0	0
Febrile neutropenia	0	3 (1.0)	0	0	0	0	0	0
Fever	10 (3.4)	3 (1.0)	0	0	10 (3.4)	5 (1.7)	0	0
Flu‐like symptoms	15 (5.1)	1 (0.3)	0	0	8 (2.7)	0	0	0
Fracture	2 (0.7)	1 (0.3)	0	0	4 (1.4)	1 (0.3)	0	0
Gastroenteritis	4 (1.4)	1 (0.3)	0	0	3 (1.0)	1 (0.3)	0	0
Gastrointestinal pain	5 (1.7)	0	0	0	4 (1.4)	1 (0.3)	0	0
GGT increased	2 (0.7)	0	0	0	0	2 (0.7)	0	0
Hyperglycaemia	1 (0.3)	0	0	0	1 (0.3)	1 (0.3)	0	0
Hypocalcaemia	8 (2.7)	1 (0.3)	0	0	9 (3.1)	0	0	0
Hypokalaemia	7 (2.4)	3 (1.0)	0	1 (0.3)	1 (0.3)	0	0	0
Hyponatraemia	1 (0.3)	0	0	0	1 (0.3)	1 (0.3)	0	0
Hypophosphataemia	4 (1.4)	0	0	0	1 (0.3)	1 (0.3)	0	0
Infections and infestations – herpes	4 (1.4)	3 (1.0)	0	0	8 (2.7)	3 (1.0)	0	0
Infections and infestations – other	31 (10.6)	8 (2.7)	1 (0.3)	0	35 (11.9)	8 (2.7)	0	0
Insomnia	13 (4.5)	0	0	0	6 (2.0)	1 (0.3)	0	0
Loss of vision	0	1 (0.3)	0	0	0	0	0	0
Lower/upper respiratory infection	81 (27.7)	24 (8.2)	1 (0.3)	1 (0.3)	87 (29.7)	33 (11.3)	2 (0.7)	1 (0.3)
Lymphocyte count decrease	3 (1.0)	1 (0.3)	0	0	5 (1.7)	0	0	0
Musculoskeletal and connective tissue disorder – other	4 (1.4)	1 (0.3)	0	0	8 (2.7)	0	0	0
Myalgia	51 (17.5)	0	0	0	43 (14.7)	0	0	0
Nausea	67 (22.9)	4 (1.4)	0	0	47 (16.0)	2 (0.7)	0	0
Neoplasms – malignant	0	4 (1.4)	0	1 (0.3)	3 (1.0)	2 (0.7)	1 (0.3)	0
Oedema limbs	8 (2.7)	1 (0.3)	0	0	16 (5.5)	0	0	0
ONJ	5 (1.7)	2 (0.7)	0	0	4 (1.4)	2 (0.7)	0	0
Pain – other	28 (9.6)	1 (0.3)	0	0	36 (12.3)	2 (0.7)	0	0
Polyp	0	0	0	0	0	1 (0.3)	0	0
Rash	40 (13.7)	4 (1.4)	0	0	49 (16.7)	2 (0.7)	0	0
Sepsis	0	3 (1.0)	2 (0.7)	1 (0.3)	1 (0.3)	5 (1.7)	0	2 (0.7)
Sinus pain	5 (1.7)	1 (0.3)	0	0	3 (1.0)	0	0	0
Skin and subcutaneous tissue disorders – other	11 (3.8)	2 (0.7)	0	0	7 (2.4)	0	0	0
Skin infection	6 (2.1)	0	0	0	4 (1.4)	1 (0.3)	0	0
Sweating	0	0	0	0	5 (1.7)	1 (0.3)	0	0
Syncope	0	1 (0.3)	0	0	2 (0.7)	1 (0.3)	0	0
Thyroid dysfunction	0	1 (0.3)	0	0	0	0	0	0
TIA	1 (0.3)	1 (0.3)	0	0	1 (0.3)	0	0	0
Urinary tract infection	17 (5.8)	2 (0.7)	0	0	20 (6.8)	2 (0.7)	0	0
Vasculitis	0	1 (0.3)	0	0	0	0	0	0
Vomiting	34 (11.6)	4 (1.4)	0	0	30 (10.2)	1 (0.3)	0	0
Weight loss	11 (3.8)	1 (0.3)	0	0	5 (1.7)	1 (0.3)	0	0
WBC decreased	11 (3.8)	1 (0.3)	0	0	25 (8.5)	0	0	0

*Note*: Grade 1–2 adverse events observed in 10% of patients in the combination lenalidomide‐vorinostat group and all Grade 3, 4 and 5 adverse events.

Abbreviations: ALP, alkaline phosphatase; ALT, alanine aminotransferase; AST, aspartate aminotransferase; CRP, C‐reactive protein; GGT, gamma‐glutamyltransferase; LFT, liver function test; ONJ, osteonecrosis of the jaw; TIA, transient ischaemic attack; WBC, white blood cell.

## DISCUSSION

Lenalidomide maintenance is an effective strategy for extending both PFS and OS compared to observation.^2^ This analysis of the Myeloma XI trial demonstrates that the addition of vorinostat to lenalidomide did not significantly improve PFS or OS, with a trend towards an impaired PFS associated with lenalidomide‐vorinostat compared to lenalidomide alone being seen in the younger patients. An interim analysis in 2017 showed that the dose of lenalidomide delivered in the lenalidomide‐vorinostat combination was reduced leading the DMEC to unblind the study with the participants receiving the combination to have the vorinostat discontinued (Supplementary Methods). At that time 69 (22.47%) patients were still receiving lenalidomide and vorinostat and so the consequence of remaining on vorinostat for those patients could not be fully determined.

While the optimum duration of maintenance has not been identified many studies have been predicated on remaining on therapy until disease progression. Coming off maintenance early, therefore, has the potential to compromise outcomes. In this study the addition of vorinostat to lenalidomide was associated with increased toxicity compared with lenalidomide alone, resulting in a median of 20 versus 30 cycles being administered respectively. Importantly, the toxicity resulted in a lower median percentage of the protocol specified dose of lenalidomide being delivered in the combination arm. Haematological toxicities, especially neutropenia and thrombocytopenia were higher in the combination arm at all grades. Grade 1–2 gastrointestinal toxicity was also higher in the combination arm, especially anorexia (13.7% vs. 6.8%) and nausea (22.9% vs. 16.0%). These low grade but long‐term toxicities appeared to hinder the capacity for patients to stay on the maintenance therapy compromising its efficacy.

Studying the impact of different induction therapy used on outcome shows that the most unfavourable outcome was seen in the patients randomised to receive thalidomide‐based induction, either CTD or CTD(a) with a PFS of 65 months with lenalidomide and 38 months with lenalidomide‐vorinostat (HR 1.50, *p* = 0.008). This appears to be associated with the lower response rates in the thalidomide‐based induction treatment arms seen previously.^8^, ^9^ Analysis by response prior to maintenance showed that patients who only achieve MR or PR had significantly shorter PFS with lenalidomide‐vorinostat than lenalidomide alone. Patients who achieved CR or VGPR to induction had similar PFS/OS with either maintenance strategy.

Inhibition of HDAC utilising panobinostat in combination with bortezomib and dexamethasone (PANORAMA2)^12^ or vorinostat in combination with bortezomib (VANTAGE)^7^ has been shown to be effective in patients with relapsed refractory myeloma, particularly in high‐risk subgroups. There was therefore a strong rationale for assessing the all‐oral combination of HDAC and lenalidomide maintenance in the up‐front setting and a prior study reporting tolerability of the lenalidomide‐vorinostat combination.^6^ However, in the Myeloma XI study, and a small study reporting only as recruitment to Myeloma XI^13^ concluded, significant toxicity was seen with HDAC inhibition and was associated with compromised delivery of the lenalidomide‐vorinostat combination. In the MUKfour trial, toxicity was also a factor limiting the utility of a vorinostat, bortezomib and dexamethasone combination in relapsed myeloma.^14^

There is a fine balance between clinical efficacy and increased toxicity especially when using drugs during the maintenance setting where tolerability and quality of life are crucial. These considerations not only apply to the use of HDAC inhibitors but have also been noted in other settings, such as the addition of clarithromycin to lenalidomide and dexamethasone for newly diagnosed patients,^15^ where although higher response rates were seen an inferior PFS was noted due to intolerability. Similarly, the checkpoint inhibitor pembrolizumab, examined in the KEYNOTE‐185 study, when added to lenalidomide and dexamethasone was associated with an excess of serious adverse events resulting in early study termination.^16^ It is not surprising, then, that the addition of a novel agent to a background of lenalidomide may not always be associated with improved outcome, especially if it impacts the ability to remain on therapy. In contrast, if the additional agent is tolerable then dual‐agent maintenance may be beneficial as shown in the FORTE trial, evaluating carfilzomib‐lenalidomide (KR) versus lenalidomide (R), which found that KR was associated with an improved PFS versus R. This difference was consistent across standard‐, high‐ and ultra‐high‐genetic‐risk groups at 3 years, suggesting it may be possible to manipulate the outcome of higher‐risk cytogenetic subgroups.^17^

In summary, this study did not reach its primary end‐point of demonstrating an improvement in PFS and OS for the lenalidomide‐vorinostat combination compared with lenalidomide alone.

## AUTHOR CONTRIBUTIONS

**Matthew W. Jenner**: Janssen – consultancy, honoraria, travel support, research funding; Takeda – consultancy, honoraria, travel support; Amgen – consultancy, honoraria, travel support; Celgene Corporation – consultancy, honoraria, research funding; Novartis – consultancy, honoraria. **Charlotte Pawlyn**: Abbvie – consultancy, Amgen – consultancy, travel support; Takeda Oncology – consultancy, travel support; Janssen – honoraria, travel support; Celgene Corporation – consultancy, honoraria, travel support, Sanofi – consultancy, honoraria. **Faith E. Davies**: Amgen – consultancy, honoraria; AbbVie – consultancy, honoraria; Takeda – consultancy, honoraria; Janssen – consultancy, honoraria; Celgene Corporation – consultancy, honoraria, research funding; Roche – consultancy, honoraria. **Tom Menzies**: Celgene Corporation, Amgen, Merck Sharp and Dohme – research funding. **Anna Hockaday**: Celgene Corporation, Amgen, Merck Sharp and Dohme – research funding. **Catherine Olivier**: Celgene Corporation, Amgen, Merck Sharp and Dohme – research funding. **John R. Jones**: Celgene Corporation – honoraria, research funding. **Kamaraj Karunanithi**: Celgene Corporation – travel support, research funding; Janssen – travel support, research funding. **Jindriska Lindsay**: Janssen – consultancy; Novartis – travel support; Takeda – honoraria, travel support; Bristol‐Myers Squibb – consultancy, travel support; Celgene Corporation – consultancy, honoraria, travel support. **Bhuvan Kishore**: Celgene Corporation, Takeda, and Janssen – consultancy, travel support, speakers’ bureau. **Gordon Cook**: Takeda – consultancy, honoraria, research funding, speakers’ bureau; Glycomimetics – consultancy, honoraria; Sanofi – consultancy, honoraria, speakers’ bureau; Celgene Corporation – consultancy, honoraria, research funding, speakers’ bureau; Janssen – consultancy, honoraria, research funding, speakers’ bureau; Bristol‐Myers Squibb – consultancy, honoraria; Amgen – consultancy, honoraria, research funding, speakers’ bureau. **Mark T. Drayson**: Abingdon Health – equity ownership, membership on an entity's board of directors or advisory committees. **Martin F. Kaiser:** Bristol‐Myers Squibb – consultancy, travel support; Chugai – consultancy; Janssen – consultancy, honoraria; Amgen – consultancy, honoraria; Takeda – consultancy, travel support; Celgene Corporation – consultancy, honoraria, research funding. **Roger G. Owen**: Takeda – honoraria, travel support; Janssen – consultancy, travel support; Celgene Corporation – consultancy, honoraria, research funding. **Walter M. Gregory**: Celgene Corporation – consultancy, research funding; Amgen, Merck Sharp and Dohme – research funding; Janssen – honoraria. **David A. Cairns**: Celgene Corporation, Amgen, Merck Sharp and Dohme – research funding. Celgene Corporation – travel support. **Gareth J. Morgan**: Janssen – research funding; Bristol‐Myers Squibb – consultancy, honoraria; Takeda – consultancy, honoraria; Celgene Corporation – consultancy, honoraria, research funding; Roche – consultancy, honoraria; Amgen – consultancy, honoraria; GSK – consultancy, honoraria; Karyopharm – consultancy, honoraria. **Graham H. Jackson**: Roche – consultancy, honoraria, speakers’ bureau; Amgen – consultancy, honoraria, speakers’ bureau; Janssen – consultancy, honoraria, speakers’ bureau; Merck Sharp and Dohme – consultancy, honoraria, speakers’ bureau; Celgene Corporation – consultancy, honoraria, travel support, research funding, speakers’ bureau; Takeda – consultancy, honoraria, travel support, research funding, speakers’ bureau.

## FUNDING INFORMATION

Financial support for the Myeloma XI trial was obtained from Cancer Research UK (CRUK; C1298/A10410). Unrestricted educational grants from Celgene Corporation and Merck Sharp and Dohme, as well as funding from Myeloma UK supported the trial co‐ordination and the laboratory studies. The funders of the study had no role in study design, data collection, data analysis, data interpretation, or writing of the report. The corresponding author had full access to all the data in the study and had final responsibility for the decision to submit for publication. This work was also supported by Core Clinical Trials Unit Infrastructure from CRUK (C7852/A25447). Charlotte Pawlyn is a CRUK Clinician Scientist (C47608/A29649).

## Supporting information


Appendix S1.

## Data Availability

De‐identified participant data will be made available when all trial primary and secondary end‐points have been met. Any requests for trial data and supporting material (data dictionary, protocol, and statistical analysis plan) will be reviewed by the trial management group in the first instance. Only requests that have a methodologically sound proposal and whose proposed use of the data has been approved by the independent TSC will be considered. Proposals should be directed to the corresponding author in the first instance; to gain access, data requestors will need to sign a data access agreement.
