# Supplementary material for: The addition of vorinostat to lenalidomide maintenance for patients with newly diagnosed multiple myeloma of all ages: results from ‘Myeloma XI’, a multicentre, open‐label, randomised, phase III trial
Source: Br J Haematol. 2022 Dec 21;201(2):267–79. doi: 10.1111/bjh.18600 (PMC10952726; doi:10.1111/bjh.18600)
Supplement: Supplementary file 1 — Appendix S1. [file BJH-201-267-s001.docx]

**The addition of vorinostat to lenalidomide maintenance for newly diagnosed multiple myeloma patients of all ages: results from Myeloma XI, a multicentre, open-label, randomised, phase 3 trial**

Contents

[Supplementary Methods 2](#_Toc117846230)

[Supplementary References 4](#_Toc117846231)

[Supplementary Table 1 – Recruitment of patients to the Lenalidomide and Lenalidomide/Vorinostat arms. 5](#_Toc117846232)

[Supplementary Table 2 – Dose and schedule of combination regimens in the trial. 8](#_Toc117846233)

[Supplementary Table 3 – Median percentage of minimum protocol dose delivered during maintenance therapy overall and by induction therapy. 9](#_Toc117846234)

[Supplementary Table 4 – Reasons for stopping maintenance therapy overall and by induction therapy. 10](#_Toc117846235)

[Supplementary Figure 1 – PFS and OS primary endpoint analysis at the time of interim analysis. 11](#_Toc117846236)

[Supplementary Figure 2 – PFS and OS primary endpoint analysis by transplant-eligibility. 12](#_Toc117846237)

[Supplementary Figure 3 – PFS and OS primary endpoint analysis by allocated induction treatment. 13](#_Toc117846238)

[Supplementary Figure 4 – PFS by response to therapy pre-maintenance 14](#_Toc117846239)

[Supplementary Figure 5 – PFS and OS primary endpoint analysis by cytogenetic risk status. 15](#_Toc117846240)

[Supplementary Figure 6 – PFS2 in the transplant-eligible and transplant-ineligible pathways. 16](#_Toc117846241)

## Supplementary Methods

***Eligibility for trial entry***

Eligible patients were aged ≥18 years and newly diagnosed with multiple myeloma requiring treatment. Exclusion criteria included previous treatment for myeloma (excluding local radiotherapy, bisphosphonates, and corticosteroids), previous or concurrent malignancies (including myelodysplastic syndromes), grade ≥2 peripheral neuropathy, acute renal failure (unresponsive to up to 72 hours of rehydration, characterized by creatinine >500 μmol/L or urine output <400 mL/day or requiring dialysis), and active or prior hepatitis C infection.

***Stratification factors***

Induction randomisation patients were stratified according to the following minimization factors: treatment centre, β_2_-microglobulin level (<3.5 mg/L, 3.5–5.5 mg/L, ≥5.5 mg/L, or unknown), haemoglobin level (<11.5 g/dL vs ≥11.5 g/dL for males; <9.5 g/dL vs ≥9.5 g/dL for females), corrected serum calcium level (<2.6 vs ≥2.6 mmol/L), serum creatinine level (<140 µmol/L vs ≥140 µmol/L), platelet count (<150 × 109/L vs ≥150 × 109/L) and centre.

Induction intensification randomised patients were stratified according to initial induction randomisation, response to initial induction treatment and treatment centre.

Maintenance randomisation patients were stratified according to treatment centre and previous randomisation group(s).

***Randomisation procedures***

All randomisations were performed at the Clinical Trials Research Unit (Leeds, UK) using a centralized automated 24-hour telephone system according to a validated minimization algorithm. Due to the nature of the intervention, patients and their physicians were aware of the treatment allocation.

***Study assessments***

Efficacy and safety measurements were performed at predefined time points; all patients in the study followed the same visit and assessment schedule. Paraprotein, serum-free light-chain analysis, and urinary light chain excretion were assessed at least every 2 months for the first 2 years and then at least every 3 months until disease progression. Response and disease progression were assessed on the basis of IMWG Uniform Response criteria (Durie*, et al* 2006, Rajkumar*, et al* 2011) and reviewed centrally by an expert panel masked to treatment allocation. Adverse events were graded according to the US National Cancer Institute Common Terminology Criteria for Adverse Events (NCI CTCAE), version 4.0. Adverse reactions were assessed at the start of each treatment cycle in patients receiving maintenance treatment. Serious adverse events were reported for all patients from the date of randomisation until 30 days after the date of disease progression except in the case of serious adverse reactions or second primary malignancies, which were collected for the duration of the trial. Second primary malignancies were reported as serious adverse events for the duration of the study (i.e., until death for each patient or when the study closes, whichever was earlier). The definition of secondary primary malignancies excluded non-melanoma skin cancers such as squamous and basal cell carcinomas of the skin.

***Study Endpoints***

For maintenance therapy comparisons, PFS was defined as the time from maintenance randomisation to the date of confirmed disease progression or death from any cause. OS was defined as the time from maintenance randomisation to the date of death from any cause. PFS2 was defined as the time from maintenance randomisation to the date of second disease progression (or start of third anti-myeloma treatment), or death from any cause. Disease progression and response were defined according to the Modified International Uniform Response Criteria (Blade*, et al* 1998, Durie*, et al* 2006, Rajkumar*, et al* 2011) and reviewed centrally by an expert panel, blinded to treatment allocation. Adverse event (AE) severity was graded according to the Common Terminology Criteria for Adverse Events (CTCAE) version 4.0.

***Cytogenetic Analysis***

Cytogenetic profiling was performed using Multiplex Ligation-dependent Probe Amplification (MLPA) and quantitative real-time PCR (qRT-PCR) (Boyle*, et al* 2015, Kaiser*, et al* 2013). Cytogenetic risk was defined as standard (no adverse lesions), high (presence of t(4;14), t(14;16), t(14;20), or del(17p), gain(1q)), or ultra-high risk (more than 1 adverse lesion) (Boyd*, et al* 2012, Sonneveld*, et al* 2016).

## Supplementary References

Blade, J., Samson, D., Reece, D., Apperley, J., Bjorkstrand, B., Gahrton, G., Gertz, M., Giralt, S., Jagannath, S. & Vesole, D. (1998) Criteria for evaluating disease response and progression in patients with multiple myeloma treated by high-dose therapy and haemopoietic stem cell transplantation. Myeloma Subcommittee of the EBMT. European Group for Blood and Marrow Transplant. *Br J Haematol,* **102,** 1115-1123.

Boyd, K.D., Ross, F.M., Chiecchio, L., Dagrada, G.P., Konn, Z.J., Tapper, W.J., Walker, B.A., Wardell, C.P., Gregory, W.M., Szubert, A.J., Bell, S.E., Child, J.A., Jackson, G.H., Davies, F.E., Morgan, G.J. & Group, N.H.O.S. (2012) A novel prognostic model in myeloma based on co-segregating adverse FISH lesions and the ISS: analysis of patients treated in the MRC Myeloma IX trial. *Leukemia,* **26,** 349-355.

Boyle, E.M., Proszek, P.Z., Kaiser, M.F., Begum, D., Dahir, N., Savola, S., Wardell, C.P., Leleu, X., Ross, F.M., Chiecchio, L., Cook, G., Drayson, M.T., Owen, R.G., Ashcroft, J.M., Jackson, G.H., Anthony Child, J., Davies, F.E., Walker, B.A. & Morgan, G.J. (2015) A molecular diagnostic approach able to detect the recurrent genetic prognostic factors typical of presenting myeloma. *Genes Chromosomes Cancer,* **54,** 91-98.

Durie, B.G., Harousseau, J.L., Miguel, J.S., Blade, J., Barlogie, B., Anderson, K., Gertz, M., Dimopoulos, M., Westin, J., Sonneveld, P., Ludwig, H., Gahrton, G., Beksac, M., Crowley, J., Belch, A., Boccadaro, M., Cavo, M., Turesson, I., Joshua, D., Vesole, D., Kyle, R., Alexanian, R., Tricot, G., Attal, M., Merlini, G., Powles, R., Richardson, P., Shimizu, K., Tosi, P., Morgan, G., Rajkumar, S.V. & International Myeloma Working, G. (2006) International uniform response criteria for multiple myeloma. *Leukemia,* **20,** 1467-1473.

Freidlin, B., Korn, E.L. & Gray, R. (2010) A general inefficacy interim monitoring rule for randomized clinical trials. *Clin Trials,* **7,** 197-208.

Kaiser, M.F., Walker, B.A., Hockley, S.L., Begum, D.B., Wardell, C.P., Gonzalez, D., Ross, F.M., Davies, F.E. & Morgan, G.J. (2013) A TC classification-based predictor for multiple myeloma using multiplexed real-time quantitative PCR. *Leukemia,* **27,** 1754-1757.

O'Brien, P.C. & Fleming, T.R. (1979) A multiple testing procedure for clinical trials. *Biometrics,* **35,** 549-556.

Rajkumar, S.V., Harousseau, J.L., Durie, B., Anderson, K.C., Dimopoulos, M., Kyle, R., Blade, J., Richardson, P., Orlowski, R., Siegel, D., Jagannath, S., Facon, T., Avet-Loiseau, H., Lonial, S., Palumbo, A., Zonder, J., Ludwig, H., Vesole, D., Sezer, O., Munshi, N.C., San Miguel, J. & International Myeloma Workshop Consensus, P. (2011) Consensus recommendations for the uniform reporting of clinical trials: report of the International Myeloma Workshop Consensus Panel 1. *Blood,* **117,** 4691-4695.

Sonneveld, P., Avet-Loiseau, H., Lonial, S., Usmani, S., Siegel, D., Anderson, K.C., Chng, W.J., Moreau, P., Attal, M., Kyle, R.A., Caers, J., Hillengass, J., San Miguel, J., van de Donk, N.W., Einsele, H., Blade, J., Durie, B.G., Goldschmidt, H., Mateos, M.V., Palumbo, A. & Orlowski, R. (2016) Treatment of Multiple Myeloma with high-risk cytogenetics: a consensus of the International Myeloma Working Group. *Blood*.

## Supplementary Table 1 – Recruitment of patients to the Lenalidomide and Lenalidomide/Vorinostat arms.

| **Centre** | **Principal Investigator(s)** | **Patients** |
| --- | --- | --- |
| Royal Stoke University Hospital, Stafford County Hospital (University Hospital North Staffordshire) | Dr Kamaraj Karunanithi, Dr Paul Revell | 24 |
| Kent and Canterbury Hospital | Dr Jindriska Lindsay | 23 |
| Birmingham Heartlands Hospital, Good Hope Hospital | Dr Bhuvan Kishore, Prof Donald Milligan | 20 |
| St James's University Hospital, Leeds | Prof Gordon Cook | 20 |
| Leicester Royal Infirmary | Dr Mamta Garg, Dr Claire Chapman | 18 |
| Nottingham City Hospital | Dr Cathy Williams, Prof Nigel Russell | 18 |
| James Cook University Hospital, Middlesbrough | Dr Raymond Dang | 17 |
| Lincoln County Hospital, Grantham and District General Hospital, Pilgrim Hospital Boston | Dr Caroline Harvey, Dr Charlotte Kallmeyer, Dr Kandeepan Saravanmuttu | 14 |
| Southampton General Hospital | Dr Matthew Jenner, Dr Alastair Smith | 13 |
| Worcestershire Royal Hospital, Alexandra Hospital Redditch, Kidderminster General Hospital | Dr Salim Shafeek | 13 |
| Royal Hallamshire Hospital, Sheffield | Prof John Snowden | 12 |
| Bristol Haematology and Oncology Centre | Dr Jenny Bird, Dr Roger Evely | 11 |
| Singleton Hospital, Swansea | Dr Hamdi Sati | 11 |
| The Christie, Manchester | Dr Samar Kulkarni, Dr Jim Cavet | 11 |
| Doncaster Royal Infirmary | Dr Joe Joseph, Dr Youssef Sorour | 10 |
| Royal Berkshire Hospital, Reading | Dr Henri Grech | 10 |
| Royal Derby Hospital | Dr David Allotey | 10 |
| Royal Marsden Hospital, London | Dr Martin Kaiser, Prof Gareth Morgan | 10 |
| Russells Hall Hospital, Dudley | Dr Craig Taylor | 10 |
| Stepping Hill Hospital, Stockport | Dr Montaser Haj | 10 |
| Warwick Hospital | Dr Carolina Arbuthnot | 10 |
| Worthing Hospital, St Richards Hospital Chichester | Dr Jamie Wilson, Dr Sarah Janes, Dr Phillip Bevan, Dr Santosh Narat | 10 |
| Medway Maritime Hospital | Dr Sarah Arnott, Dr Vijay Dhanapal, Dr Vivienne Andrews | 9 |
| Royal Oldham Hospital | Dr Hayley Greenfield | 9 |
| Cheltenham General Hospital, Gloucestershire Royal Hospital | Dr Sally Chown | 8 |
| Diana Princess of Wales Hospital, Grimsby | Dr Susan Levison-Keating, Dr Sanjeev Jalihal, Dr Hannah Ciepluch | 8 |
| Royal Blackburn Hospital | Dr Malgorzata Rokicka, Dr Jagdish Adiyodi | 8 |
| Royal Bournemouth Hospital | Dr Rachel Hall | 8 |
| Royal Devon and Exeter Hospital | Dr Tony Todd, Dr Claudius Rudin | 8 |
| Royal Gwent Hospital, Newport | Dr Helen Jackson | 8 |
| Sandwell General Hospital, West Bromwich | Dr Farooq Wandroo | 8 |
| Stoke Mandeville Hospital, Wycombe Hospital | Dr Robin Aitchison | 8 |
| Blackpool Victoria Hospital | Dr Mark Grey, Dr Marian Paul Macheta | 7 |
| Maidstone Hospital, Tunbridge Wells Hospital | Dr Don Gillett, Dr Lalita Banerjee | 7 |
| Royal Cornwall Hospital, Truro | Dr Julie Blundell | 7 |
| Salisbury District Hospital | Dr Jonathan Cullis | 7 |
| Southmead Hospital, Bristol (Frenchay) | Dr Alastair Whiteway | 7 |
| University Hospital of Wales Cardiff, Llandough Hospital | Dr Ceri Bygrave, Dr Christopher Fegan, Dr Belinda Austin | 7 |
| Western General Hospital, Edinburgh | Dr Huw Roddie | 7 |
| Calderdale Royal Hospital, Huddersfield Royal Infirmary | Dr Kate Rothwell, Dr Sylvia Feyler | 6 |
| Chesterfield Royal Hospital | Dr Peter Toth, Dr Emma Welch | 6 |
| Derriford Hospital, Plymouth | Dr Hannah Hunter | 6 |
| Freeman Hospital, Newcastle | Prof Graham Jackson | 6 |
| Ipswich Hospital | Dr Isobel Chalmers | 6 |
| Kettering General Hospital | Dr Mark Kwan | 6 |
| New Cross Hospital, Wolverhampton | Dr Supratik Basu | 6 |
| Sunderland Royal Hospital | Dr Victoria Hervey, Dr Scott Marshall, Dr Simon Lyons | 6 |
| Wythenshawe Hospital, Manchester | Dr Simon Watt | 6 |
| York Hospital, Scarborough General Hospital | Dr Laura Munro, Dr Haz Sayala | 6 |
| Aberdeen Royal Infirmary | Dr Jane Tighe | 5 |
| Countess of Chester Hospital | Dr Gillian Brearton, Dr Salah Tueger | 5 |
| Dorset County Hospital | Dr Dietman Hofer, Dr Akeel Moosa | 5 |
| Eastbourne Hospital, Conquest Hospital | Dr Sunil Gupta, Dr Simon Weston-Smith, Dr Satyajit Sahu | 5 |
| King's Mill Hospital, Sutton-in-Ashfield | Dr Tim Moorby, Dr Rowena Faulkner | 5 |
| Manchester Royal Infirmary, Trafford General Hospital | Dr Alberto Rocci, Dr Eleni Tholouli, Dr John Alderson, Dr Simon Gibbs | 5 |
| Poole Hospital | Dr Ram Jayaprakash, Dr Fergus Jacki | 5 |
| Queen Elizabeth Hospital, Birmingham | Dr Mark Cook | 5 |
| Royal Lancaster Infirmary | Dr David Howarth | 5 |
| Royal Preston Hospital | Dr Mark Grey, Dr Frederick Kanyike, Dr Maqsood Punekar | 5 |
| University Hospital Aintree | Dr Lynny Yung, Dr Barbara Hammer | 5 |
| University Hospital Coventry | Dr Beth Harrison, Dr Syed Bokhari | 5 |
| Colchester General Hospital | Dr Michael Hamblin, Dr Sudhakaran Makkuni | 4 |
| Hereford County Hospital | Dr Lisa Robinson | 4 |
| Queen's Hospital, Romford | Dr Sandra Hassan, Dr Biju Krishnan, Dr Jane Stevens | 4 |
| Royal Bolton Hospital | Dr Chetan Patalappa, Dr Suzanne Roberts, Dr Mark Grey, Dr Claire Barnes | 4 |
| Arrowe Park, Wirral | Dr Ranjit Dasgupta, Dr Nauman Butt | 3 |
| Beatson Oncology Centre, Glasgow | Dr Richard Soutar | 3 |
| Borders General Hospital, Melrose | Dr Jenny Buxton, Dr Srivnivasa Dasari, Dr John Tucker, Dr Ashok Okhandiar | 3 |
| Ninewells Hospital Dundee, Perth Royal Infirmary | Dr Duncan Gowans | 3 |
| Norfolk and Norwich University Hospital | Dr Martin Auger, Dr Kristian Bowles | 3 |
| North Devon District Hospital, Barnstaple | Dr Paul Kerr, Dr Malcolm Hamilton | 3 |
| Royal Liverpool Hospital | Dr Stephen Hawkins, Prof Patrick Chu | 3 |
| Scunthorpe General Hospital | Dr Sanjeev Jalihal | 3 |
| Castle Hill Hospital, Hull | Dr David Allsup, Dr Haz Sayala | 2 |
| Darent Valley Hospital | Dr Tariq Shafi, Dr Anil Kamat | 2 |
| Harrogate District Hospital | Dr Claire Hall | 2 |
| Monklands Hospital, Hairmyres Hospital, Wishaw General Hospital | Dr Iain Singer | 2 |
| Nevill Hall Hospital, Abergavenny | Dr Nilima Parry-Jones | 2 |
| Torbay Hospital, Torquay | Dr Heather Eve, Dr Deborah Turner | 2 |
| Addenbrookes Hospital, Cambridge | Dr Jenny Craig, Dr Charles Crawley | 1 |
| George Eliot Hospital, Nuneaton | Dr Mekkali Narayanan | 1 |
| James Paget Hospital, Great Yarmouth | Dr Cesar Gomez, Dr Shalal Sadullah | 1 |
| Rotherham General Hospital | Dr Richard Went, Dr Helen Barker | 1 |
| Victoria Hospital Kirkcaldy | Dr Lorna McClintock | 1 |
| Ysbyty Gwynedd, Bangor | Dr Sally Evans, Dr Melinda Hamilton, Dr David Edwards | 1 |

## Supplementary Table 2 – Dose and schedule of combination regimens in the trial.

| Regimen | Dose and schedule | |
| --- | --- | --- |
| CRD  (cyclophosphamide, lenalidomide, dexamethasone) | C: 500 mg po on days 1, 8  R: 25 mg daily po on days 1-21  D: 40 mg daily po on days 1-4, 12-15 | Cycles repeat every 28 days for at least 4 cycles and until maximum response achieved. |
| CTD  (cyclophosphamide, thalidomide, dexamethasone) | C: 500 mg po on days 1, 8, 15  T: 100 mg daily po for 3 weeks,  increasing to 200 mg daily po  D: 40 mg daily po on days 1-4, 12-15 | Cycles repeat every 21 days for at least 4 cycles and until maximum response achieved. |
| CRDa  (attenuated-dose CRD) | C: 500 mg po on days 1, 8 R: 25 mg daily po on days 1–21 D: 20 mg daily po on days 1–4, 15–18 | Cycles repeat every 28 days for ≥ 6 cycles and until maximum response or intolerance |
| CTDa  (attenuated-dose CTD) | C: 500 mg po on days 1, 8, 15, 22 T: 50 mg daily po for 4 weeks, increasing in 50 mg increments every 4 weeks to 200 mg daily po D: 20 mg daily po on days 1–4, 15–18 | Cycles repeat every 28 days for ≥ 6 cycles and until maximum response or intolerance |
| CVD (cyclophosphamide, bortezomib, dexamethasone) | C: 500 mg daily po on days 1, 8, 15 V: 1.3 mg/m^2^ sc or iv on days 1, 4, 8, 11 D: 20 mg daily po on days 1, 2, 4, 5, 8, 9, 11, 12 | Cycles repeat every 21 days until maximum response or intolerance (maximum 8 cycles);  if CR is achieved, continue treatment for a maximum of 2 additional cycles |
| Lenalidomide maintenance* | 10 mg daily po on days 1–21 | Cycles repeat every 28 days and continue, in the absence of toxicity, until PD |
| Combination lenalidomide-vorinostat maintenance* | R: 10 mg daily po on days 1–21  Vorinostat: 300 mg daily po on days 1–7 and 15–21 | Cycles repeat every 28 days and continue, in the absence of toxicity, until PD |

* Patients were accrued to the maintenance randomization between January 13, 2011 and August 11, 2017. Patients were initially randomized in a 1:1 ratio, using minimization with a bias element of 80%, to either R 25 mg/day (po on days 1–21 of each 28-day cycle) or observation, stratified by induction and intensification treatment. Following a protocol amendment on September 14, 2011 and after accrual of 442 patients under protocol versions 2·0–4·0, patients were randomized in a 1:1:1 ratio to R 10 mg/day (po on days 1–21 of each 28-day cycle), R + Z, or observation. Following a further protocol amendment on June 28, 2013 and after accrual of 615 further patients under protocol version 5·0, patients were randomized in a 2:1 ratio to R 10 mg/day or observation; R plus vorinostat was discontinued under protocol version 6·0. These changes were made to add research questions to this adaptive design study. Abbreviations: a, attenuated-dose; C, cyclophosphamide; CR, complete response; D, dexamethasone; iv, intravenously; PD, disease progression; po, orally; R, lenalidomide; R+Z, combination lenalidomide-vorinostat; sc, subcutaneously; T, thalidomide; V, bortezomib.

## Supplementary Table 3 – Median percentage of minimum protocol dose delivered during maintenance therapy overall and by induction therapy.

|  | **Lenalidomide** | | | | | **Combination lenalidomide-vorinostat** | | | | |
| --- | --- | --- | --- | --- | --- | --- | --- | --- | --- | --- |
|  | **Total** | **Induction therapy** | | | | **Total** | **Induction therapy** | | | |
|  |  | **CTD** | **CRD** | **CTDa** | **CRDa** |  | **CTD** | **CRD** | **CTDa** | **CRDa** |
|  | **(n=307)** | **(n=94)** | **(n=105)** | **(n=49)** | **(n=59)** | **(n=307)** | **(n=93)** | **(n=103)** | **(n=50)** | **(n=61)** |
| **Percentage of minimum protocol dose delivered (median, IQR)** |  |  |  |  |  |  |  |  |  |  |
| **Lenalidomide** | 78.2%  (43.1%–100.0%) | 59.5%  (20.8%–94.4%) | 85.7%  (49.9%–99.7%) | 72.8%  (33.2%–100.0%) | 93.9%  (51.1%–100.0%) | 54.6%  (18.9%–93.8%) | 49.9%  (18.3%–91.8%) | 59.4%  (22.6%–92.7%) | 51.1%  (12.5%–94.7%) | 56.8%  (20.0%–96.7%) |
| **Vorinostat** |  |  |  |  |  | 30.2%  (6.1%–67.7%) | 28.9%  (4.8%–66.7%) | 28.6%  (6.6%–69.5%) | 42.3%  (12.5%–79.4%) | 42.7%  (16.3%–73.0%) |
|  |  |  |  |  |  |  |  |  |  |  |

IQR, inter-quartile range.

## Supplementary Table 4 – Reasons for stopping maintenance therapy overall and by induction therapy.

|  | **Lenalidomide** | | | | | **Combination lenalidomide-vorinostat** | | | | |
| --- | --- | --- | --- | --- | --- | --- | --- | --- | --- | --- |
|  | **Total** | **Induction therapy** | | | | **Total** | **Induction therapy** | | | |
|  |  | **CTD** | **CRD** | **CTDa** | **CRDa** |  | **CTD** | **CRD** | **CTDa** | **CRDa** |
|  | **(n=307)** | **(n=94)** | **(n=105)** | **(n=49)** | **(n=59)** | **(n=307)** | **(n=93)** | **(n=103)** | **(n=50)** | **(n=61)** |
| **Patients continuing maintenance therapy** N, (%) | 91 ( 29.6%) | 32 ( 34.0%) | 39 ( 37.1%) | 11 ( 22.4%) | 9 ( 15.3%) | 60 ( 19.5%) | 15 ( 16.1%) | 27 ( 26.2%) | 6 ( 12.0%) | 12 ( 19.7%) |
| **Reasons for stopping maintenance therapy*** N, (%) |  |  |  |  |  |  |  |  |  |  |
| **Disease progression** | 125 (40.7%) | 29 ( 30.9%) | 40 ( 38.1%) | 23 ( 46.9%) | 33 ( 55.9%) | 109 (35.5%) | 33 ( 35.5%) | 35 ( 34.0%) | 18 ( 36.0%) | 23 ( 37.7%) |
| **Patient died** |  |  |  |  |  | 5 ( 1.6%) | 1 ( 1.1%) | 1 ( 1.0%) | 3 ( 6.0%) |  |
| **Patient choice** | 16 ( 5.2%) | 10 ( 10.6%) | 3 ( 2.9%) | 3 ( 6.1%) |  | 22 ( 7.2%) | 9 ( 9.7%) | 7 ( 6.8%) | 1 ( 2.0%) | 5 ( 8.2%) |
| **Unacceptable toxicity** | 38 ( 12.4%) | 14 ( 14.9%) | 7 ( 6.7%) | 7 ( 14.3%) | 10 ( 16.9%) | 78 ( 25.4%) | 25 ( 26.9%) | 23 ( 22.3%) | 17 ( 34.0%) | 13 ( 21.3%) |
| **Secondary malignancy** | 5 ( 1.6%) |  | 2 ( 1.9%) | 1 ( 2.0%) | 2 ( 3.4%) | 5 ( 1.6%) | 2 ( 2.2%) | 3 ( 2.9%) |  |  |
| **Clinician choice** | 15 ( 4.9%) | 6 ( 6.4%) | 6 ( 5.7%) | 2 ( 4.1%) | 1 ( 1.7%) | 8 ( 2.6%) | 3 ( 3.2%) | 1 ( 1.0%) | 1 ( 2.0%) | 3 ( 4.9%) |
| **Other** | 17 ( 5.5%) | 3 ( 3.2%) | 8 ( 7.6%) | 2 ( 4.1%) | 4 ( 6.8%) | 20 ( 6.5%) | 5 ( 5.4%) | 6 ( 5.8%) | 4 ( 8.0%) | 5 ( 8.2%) |

## Supplementary Figure 1 – PFS and OS primary endpoint analysis at the time of interim analysis.

(A) PFS. (B) OS.

PFS, progression‐free survival; OS, overall survival; R, lenalidomide; R+Z, combination lenalidomide-vorinostat; CI, confidence interval.

| A | B |
| --- | --- |
| 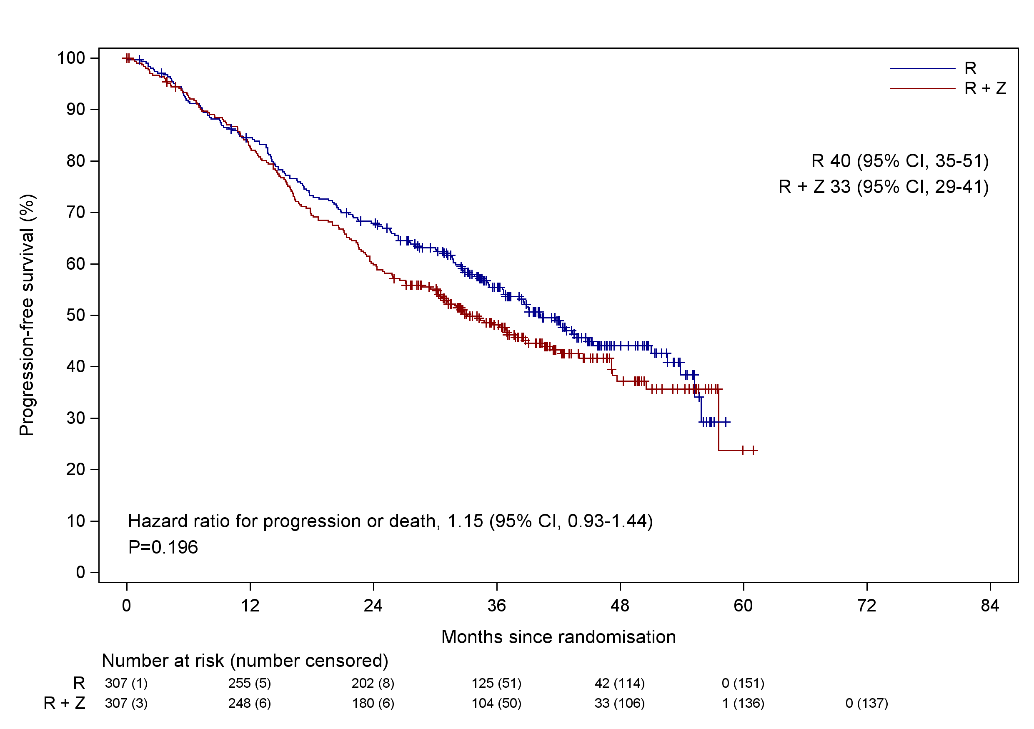 | 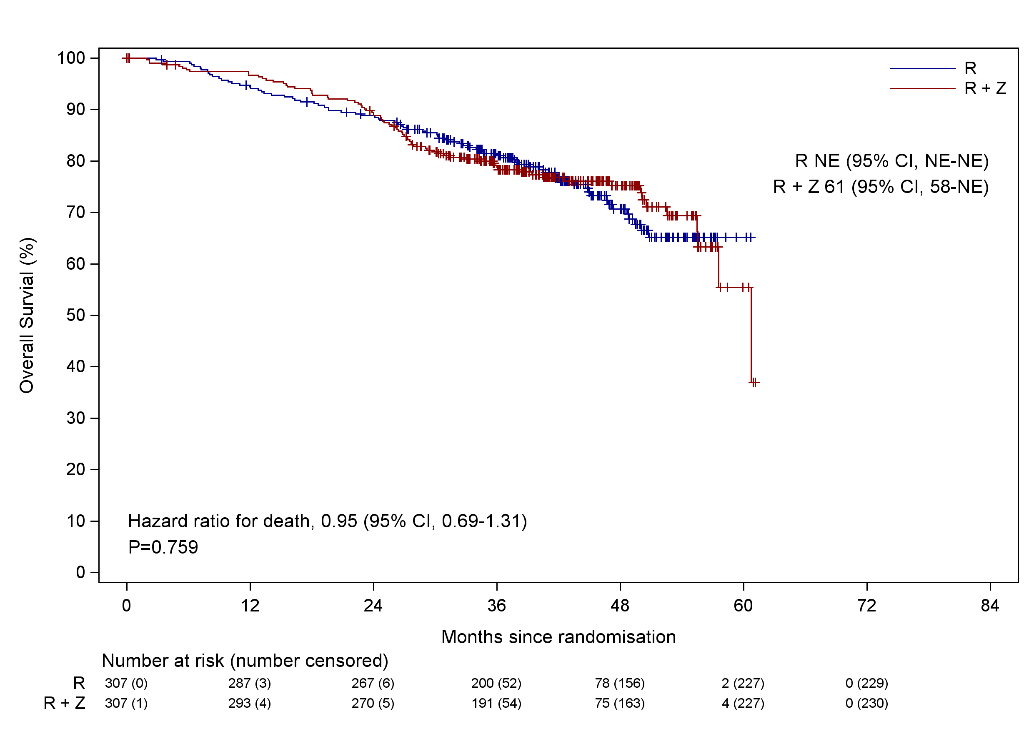 |

## Supplementary Figure 2 – PFS and OS primary endpoint analysis by transplant-eligibility.

(A) PFS: TE. (B) OS: TE. (C) PFS: TNE. (D) OS: TNE.

PFS, progression‐free survival; OS, overall survival; TE, transplant-eligible; TNE, transplant-ineligible; R, lenalidomide; R+Z, combination lenalidomide-vorinostat; CI, confidence interval.

| A | B |
| --- | --- |
| 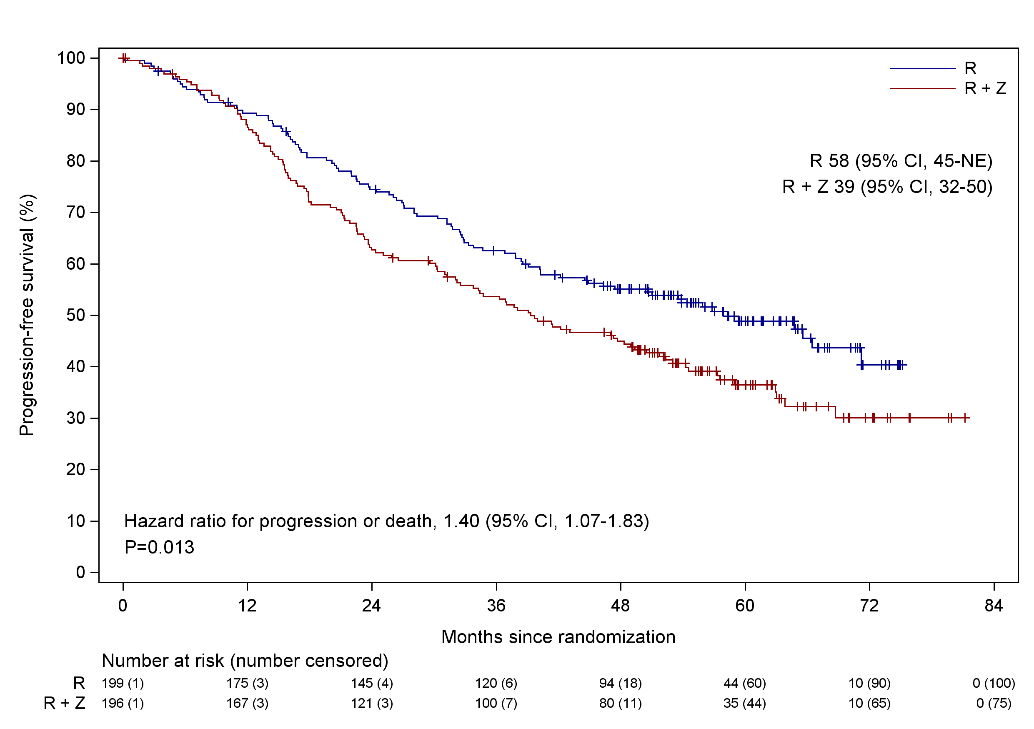 | 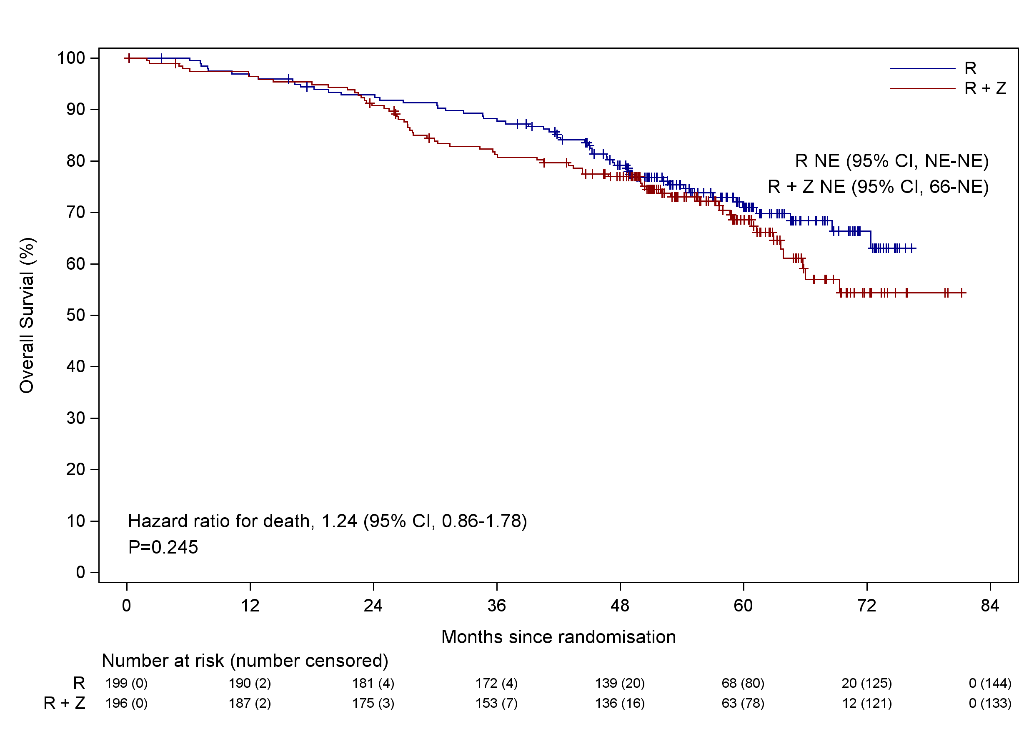 |
| C | D |
| 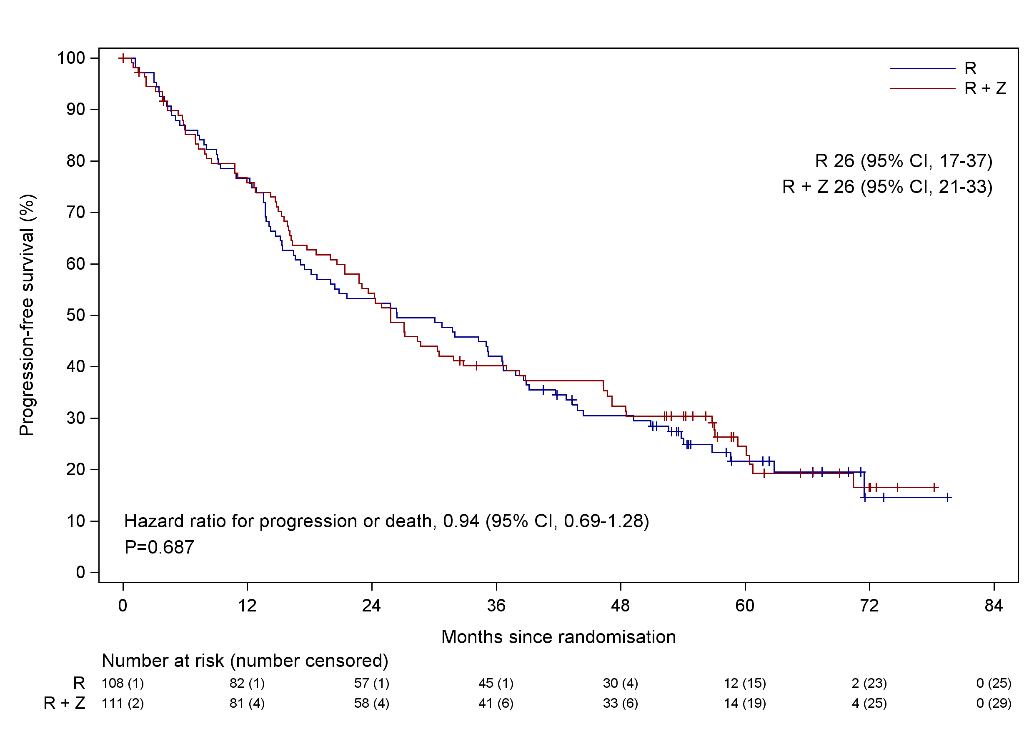 | 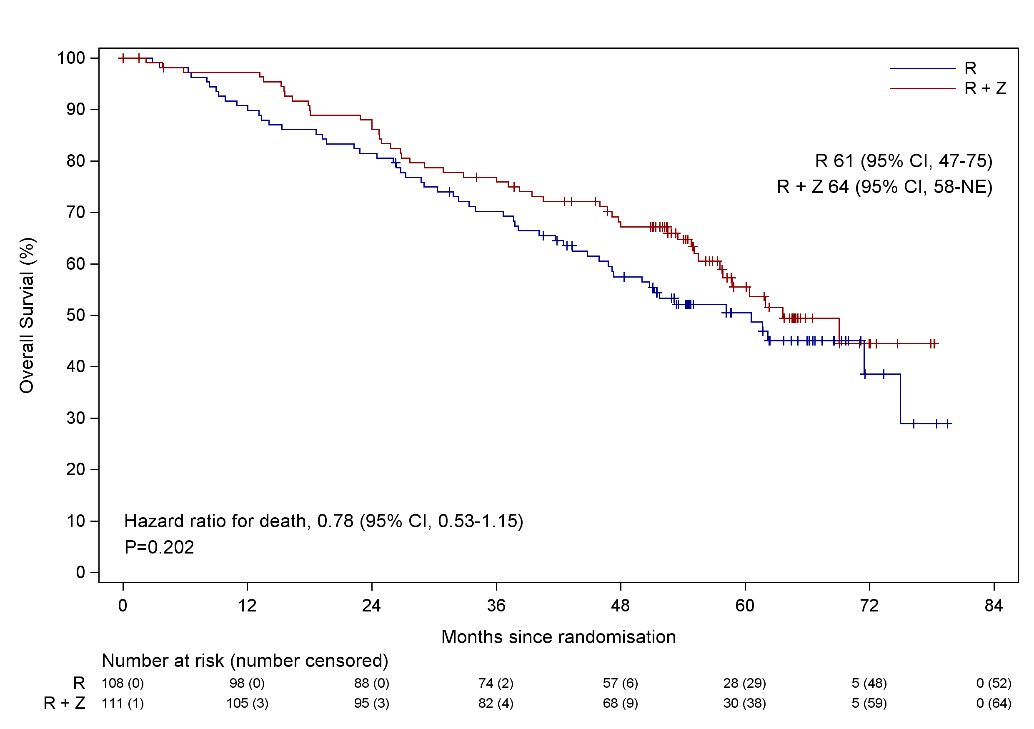 |

## Supplementary Figure 3 – PFS and OS primary endpoint analysis by allocated induction treatment.

(A) PFS: CTD(a). (B) OS: CTD(a). (C) PFS: CRD(a). (D) OS: CRD(a).

PFS, progression‐free survival; OS, overall survival; CTD(a), cyclophosphamide, thalidomide, dexamethasone (attenuated); CRD(a), cyclophosphamide, lenalidomide, dexamethasone (attenuated); R, lenalidomide; R+Z, combination lenalidomide-vorinostat; CI, confidence interval.

| A | B |
| --- | --- |
| 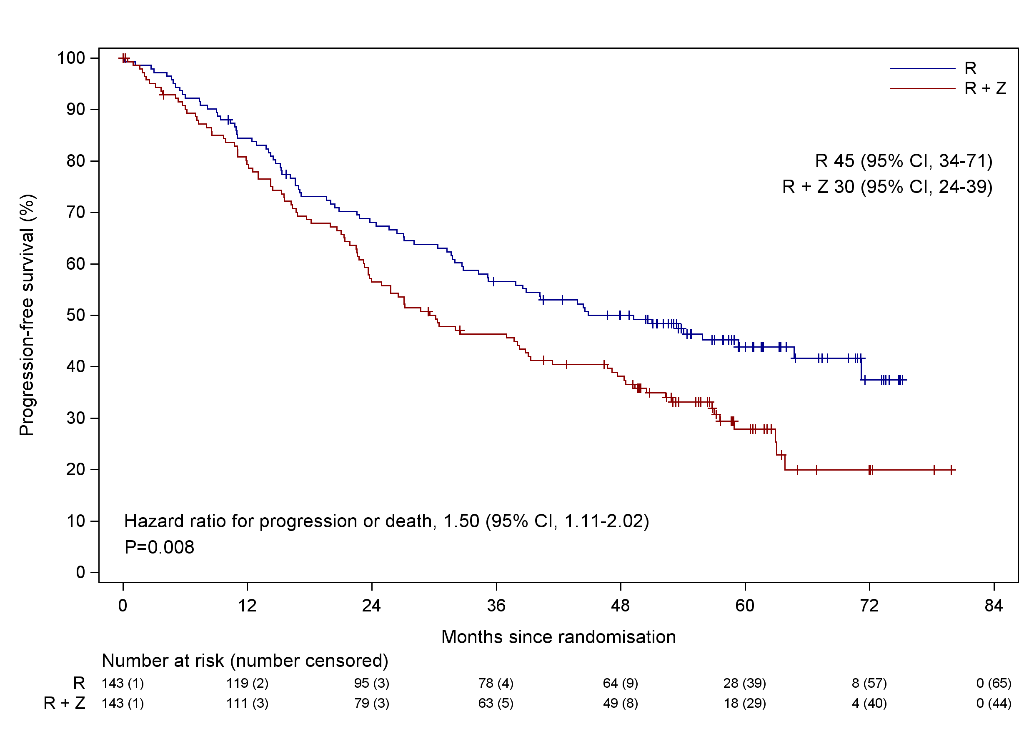 | 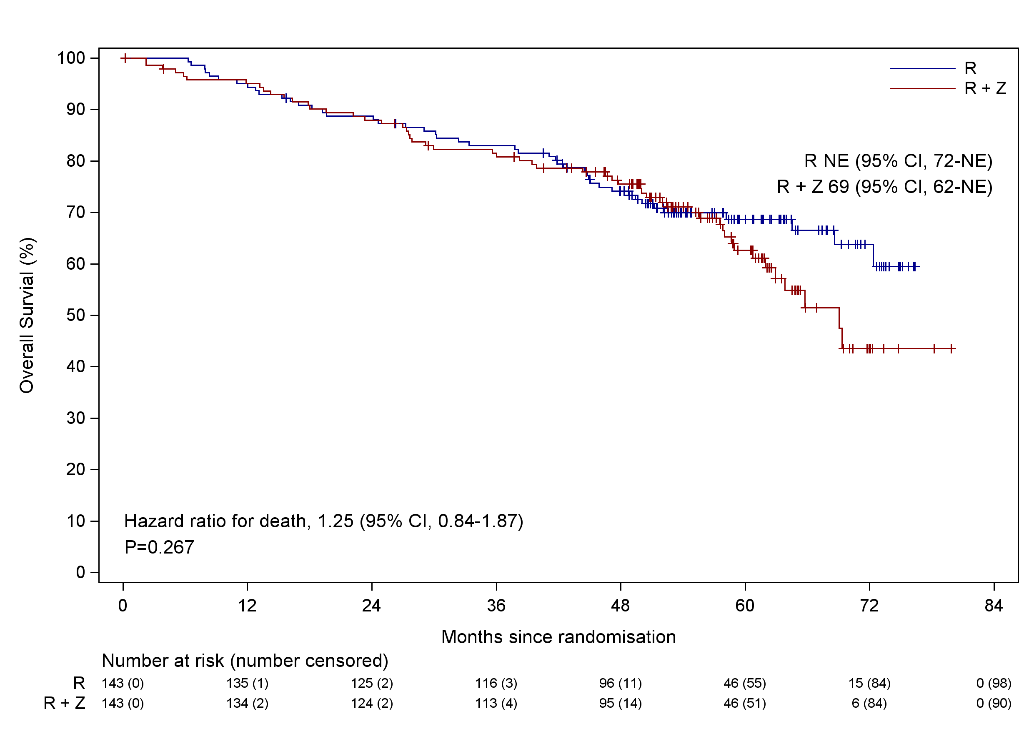 |
| C | D |
| 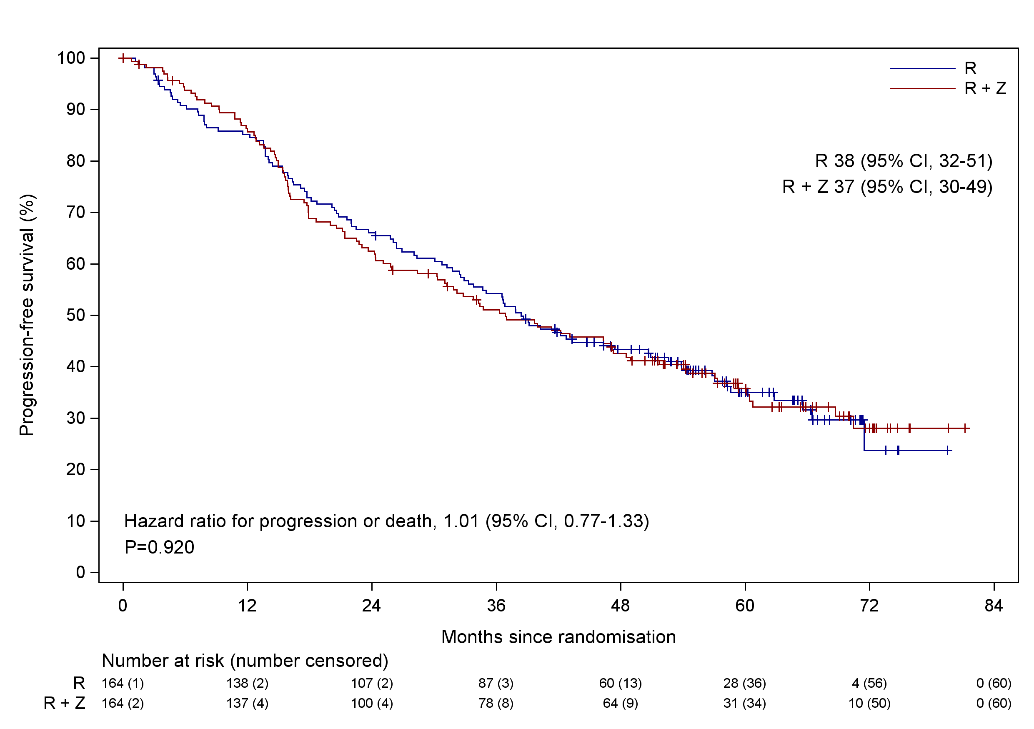 | 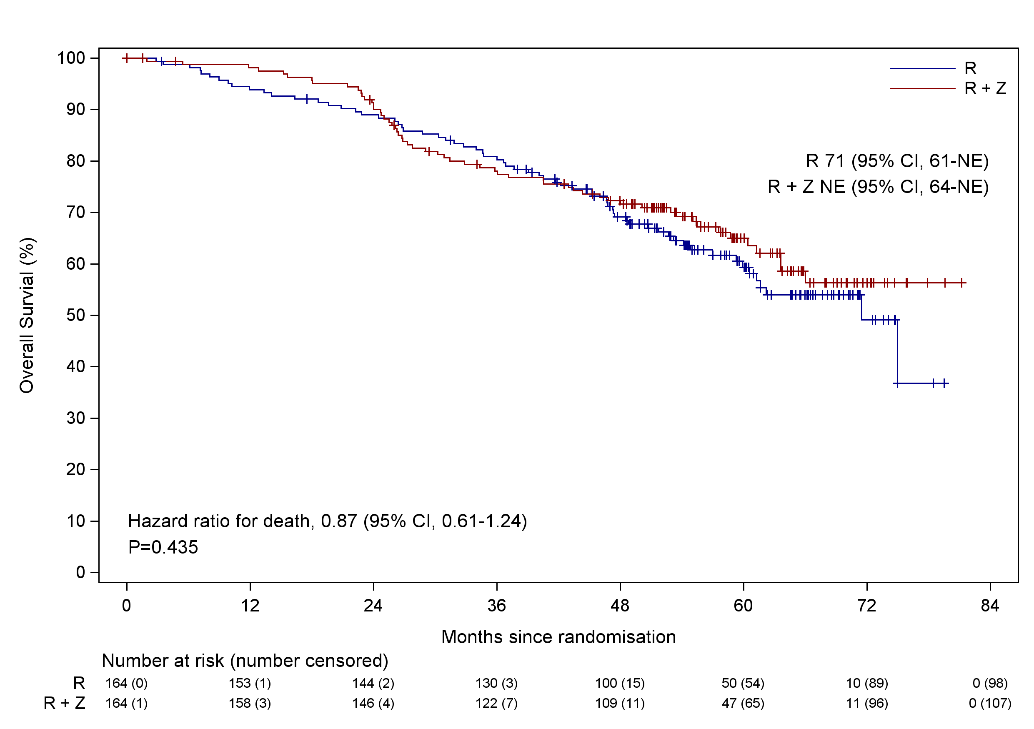 |

## Supplementary Figure 4 – PFS by response to therapy pre-maintenance

(A) CR/VGPR, (B) PR/MR

PFS, progression‐free survival; R, lenalidomide; R+Z, combination lenalidomide-vorinostat; CI, confidence interval.

| A | B |
| --- | --- |
| 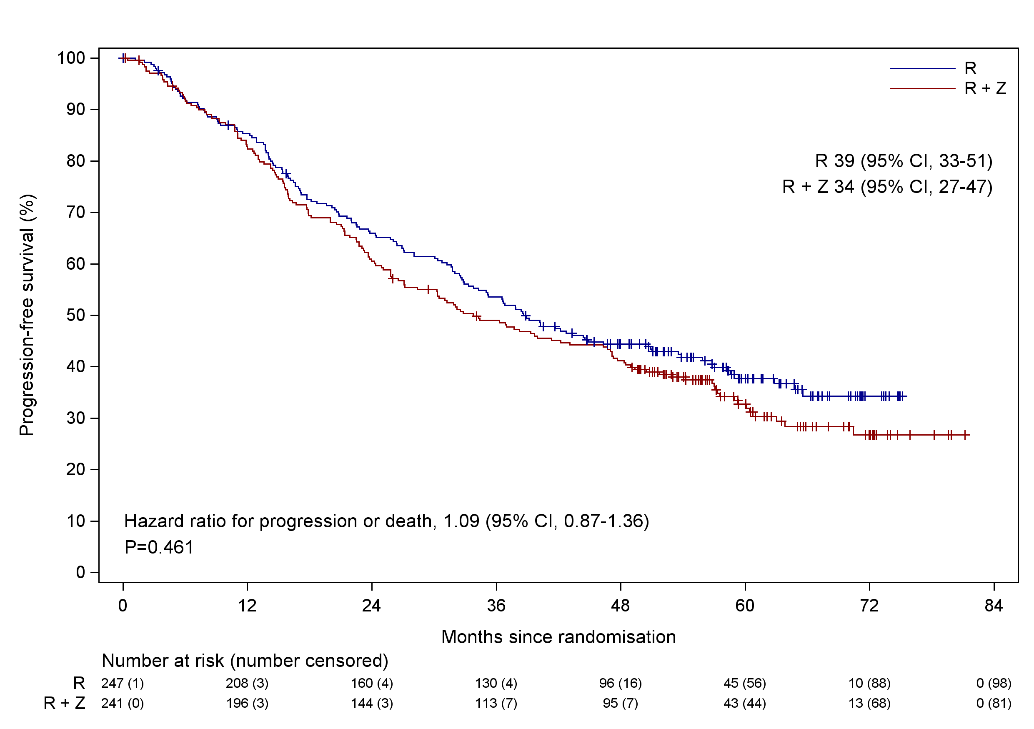 | 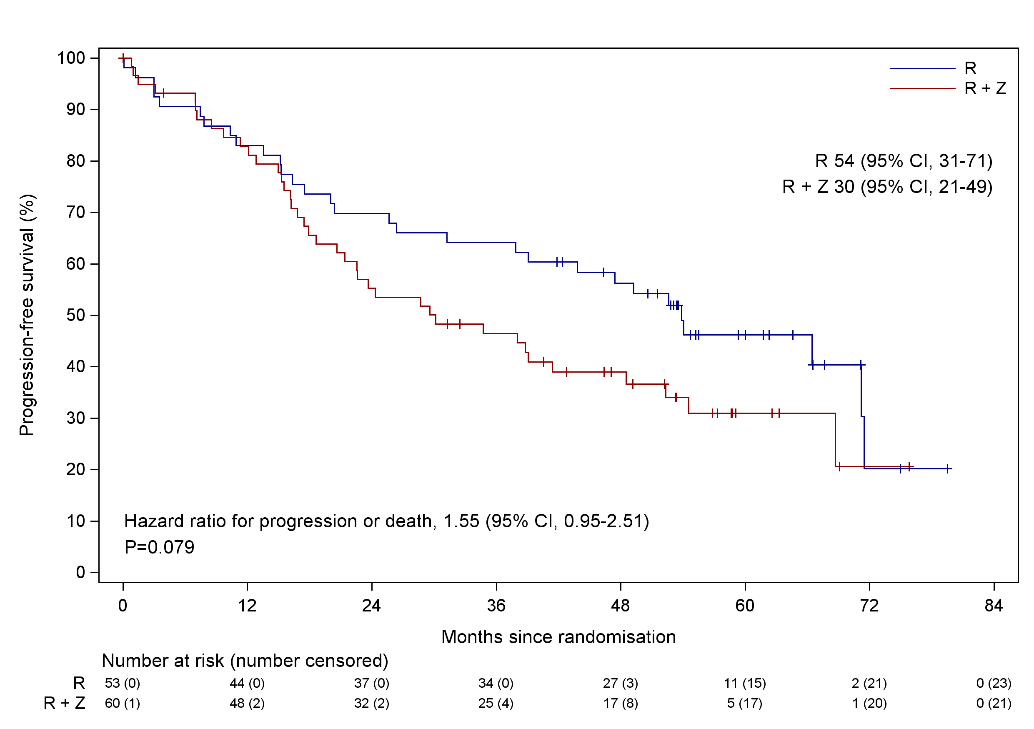 |
|  |  |
|  |  |

## Supplementary Figure 5 – PFS and OS primary endpoint analysis by cytogenetic risk status.

(A) PFS: SR. (B) OS: SR. (C) PFS: HR. (D) OS: HR. (E) PFS: UHiR. (F) OS: UHiR; R, lenalidomide; R+Z, combination lenalidomide-vorinostat; CI, confidence interval.

| A | B |
| --- | --- |
| 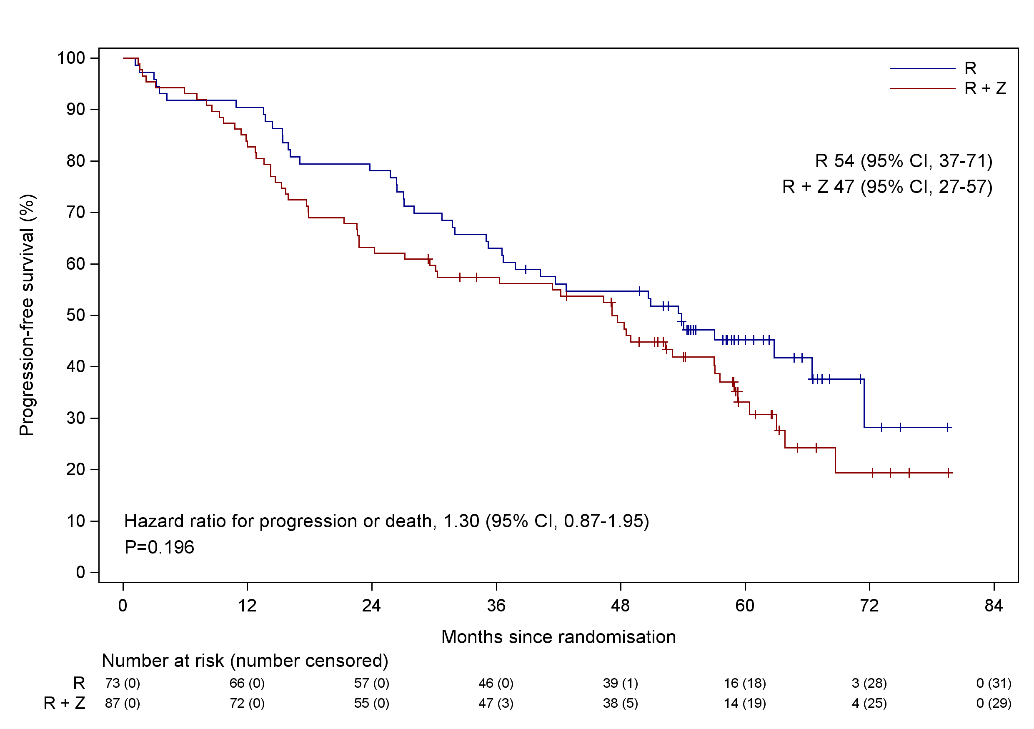 | 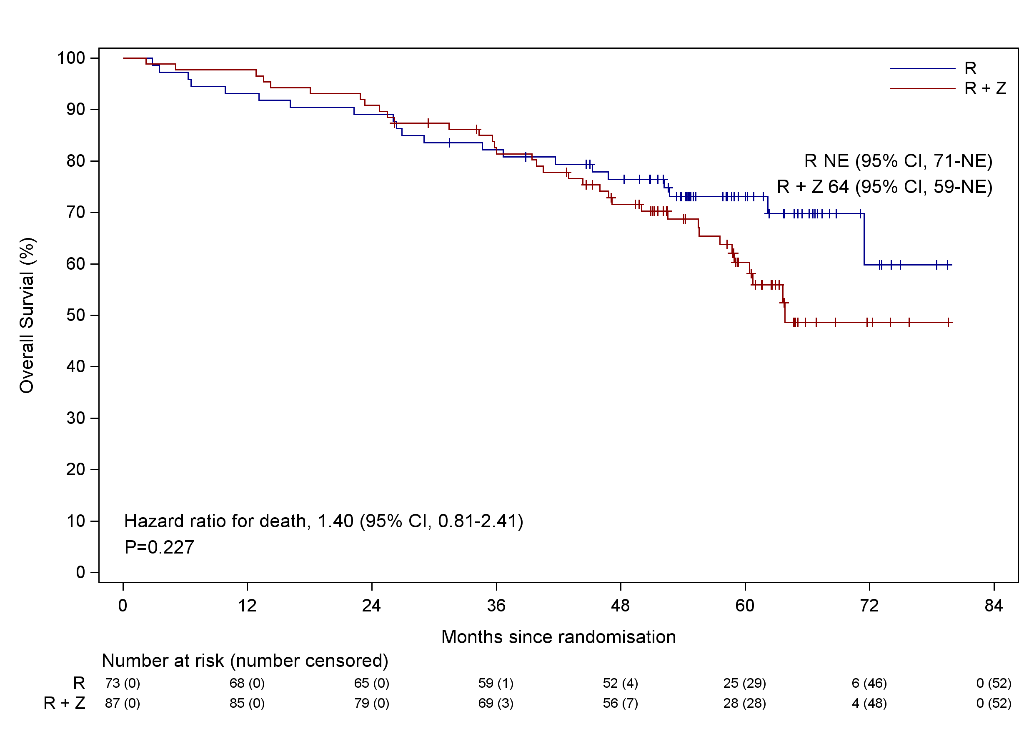 |
| C | D |
| 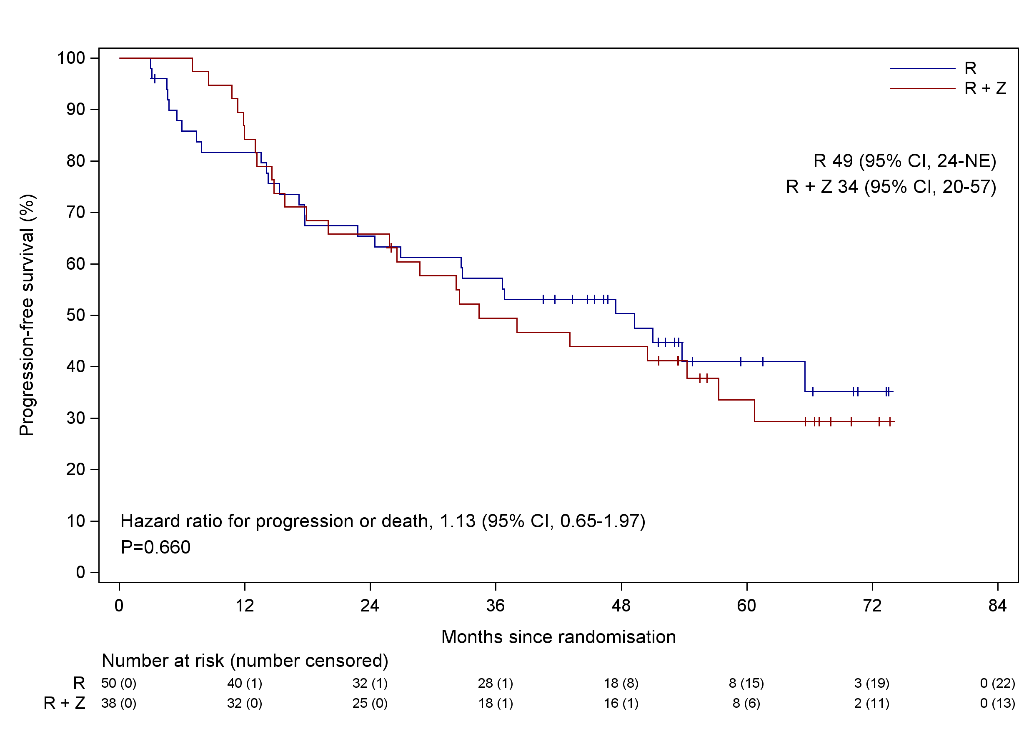 | 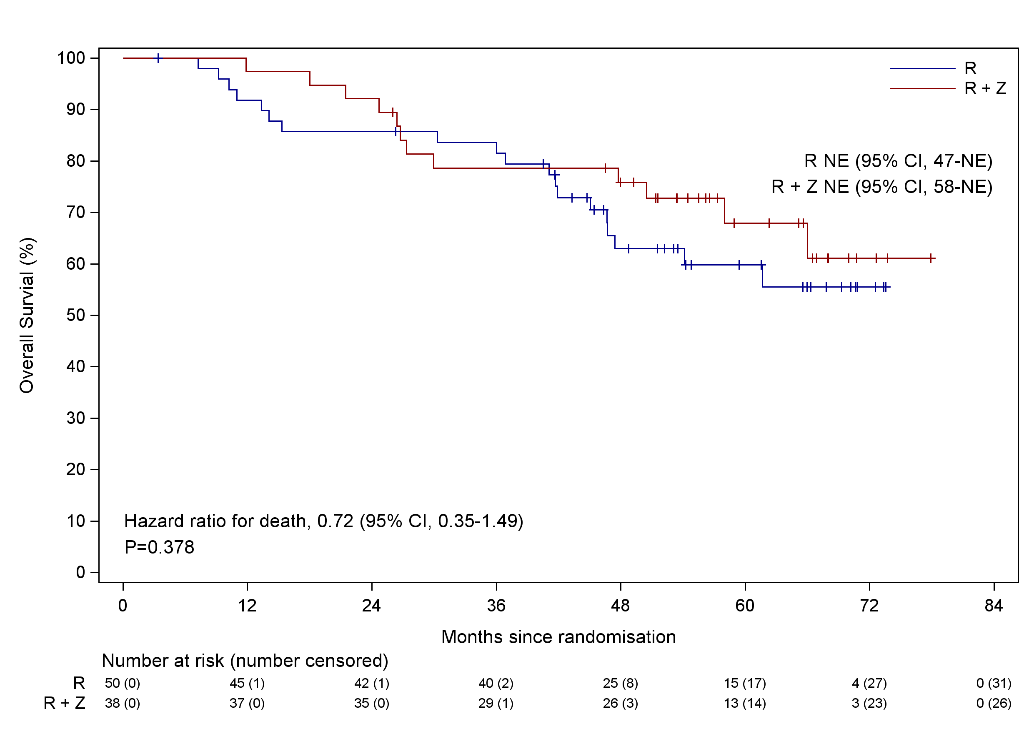 |
| E | F |
| 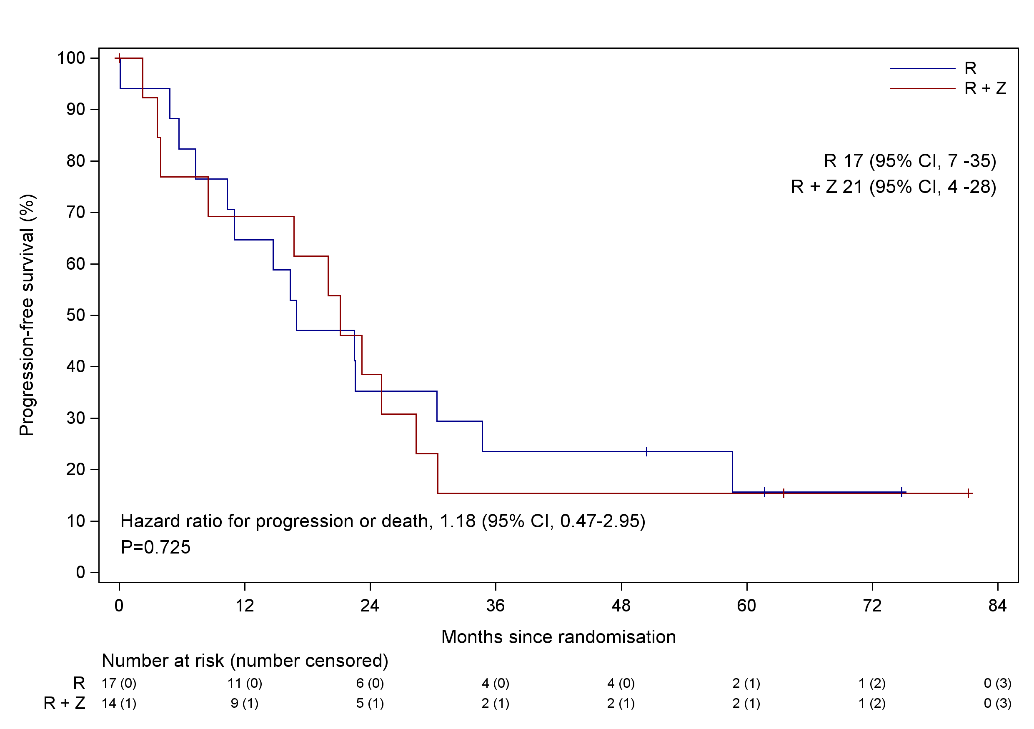 | 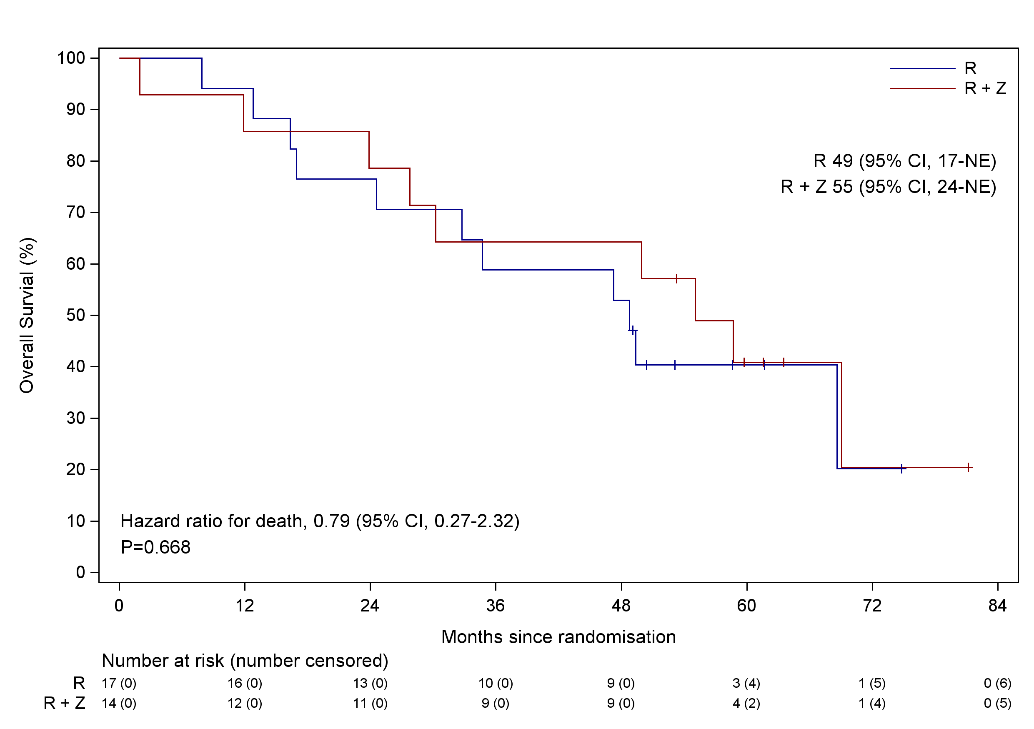 |

## Supplementary Figure 6 – PFS2 in the transplant-eligible and transplant-ineligible pathways.

(A) TE. (B) TNE.

PFS2, progression‐free survival 2; TE, transplant-eligible; TNE, transplant-ineligible; R, lenalidomide; R+Z, combination lenalidomide-vorinostat; CI, confidence interval.

| A | B |
| --- | --- |
| 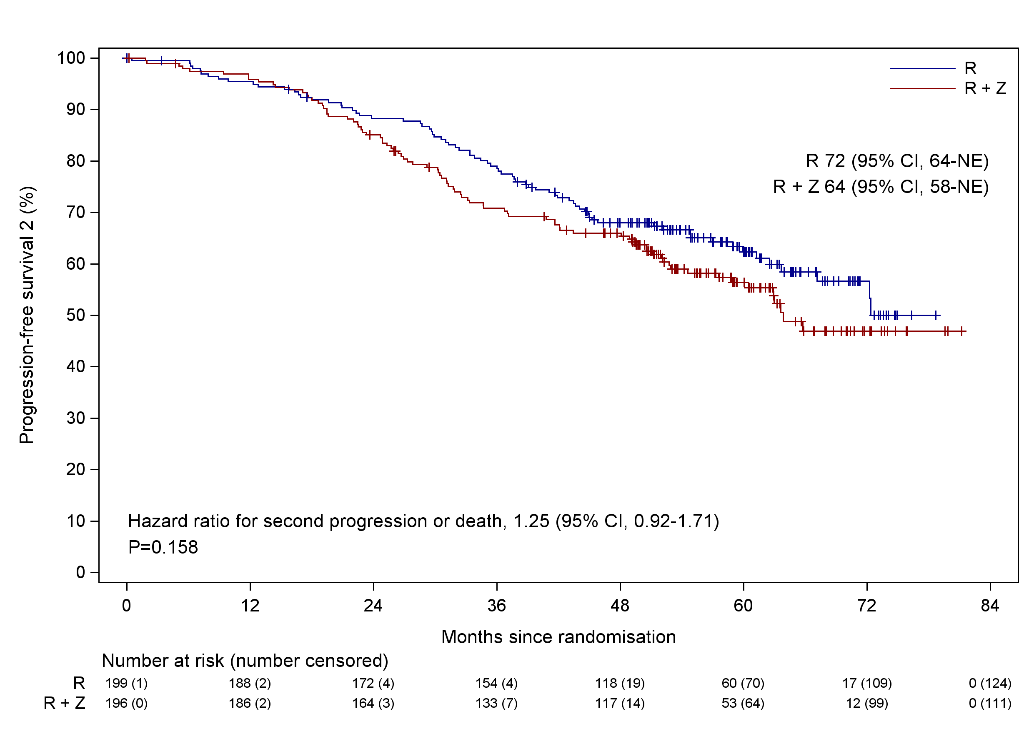 | 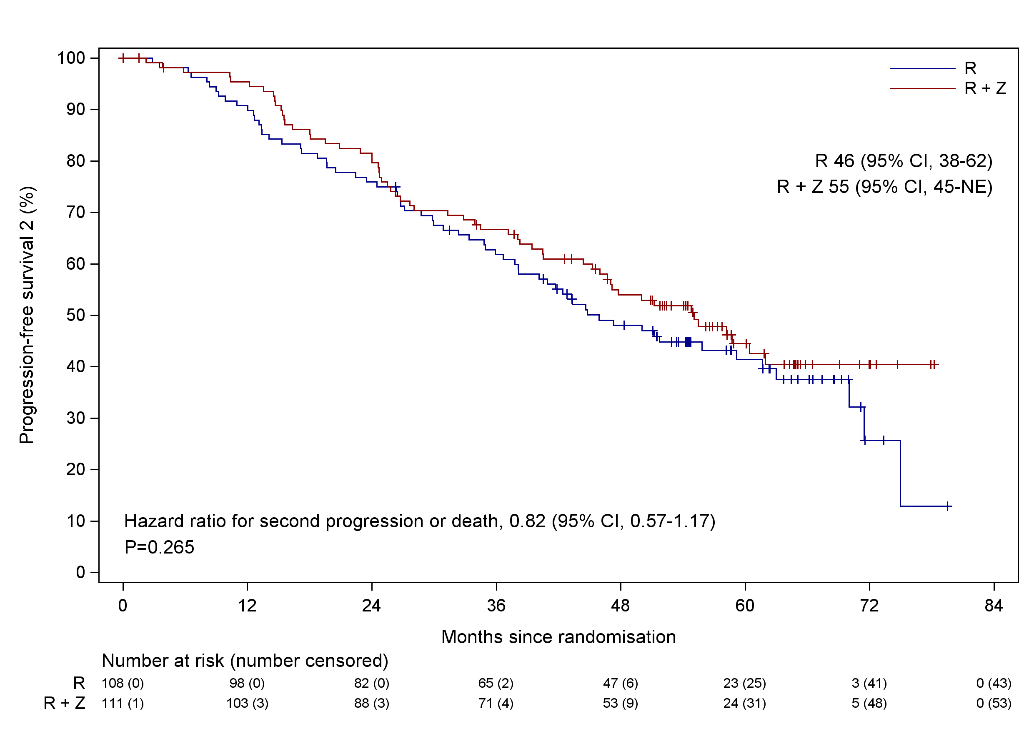 |
